# Thermal growth in solar water pump using Prandtl–Eyring hybrid nanofluid: a solar energy application

**DOI:** 10.1038/s41598-021-98103-8

**Published:** 2021-09-21

**Authors:** Wasim Jamshed, Nor Ain Azeany Mohd Nasir, Siti Suzilliana Putri Mohamed Isa, Rabia Safdar, Faisal Shahzad, Kottakkaran Sooppy Nisar, Mohamed R. Eid, Abdel-Haleem Abdel-Aty, I. S. Yahia

**Affiliations:** 1 Department of Mathematics, Capital University of Science and Technology (CUST), Islamabad, 44000 Pakistan; 2 Department of Mathematics, Centre for Defence Foundation Studies, Universiti Pertahanan Nasional Malaysia, Kem Sungai Besi, 57000 Kuala Lumpur, Malaysia; 3 Institute for Mathematical Research, Universiti Putra Malaysia, 43400 UPM Serdang, Selangor Darul Ehsan Malaysia; 4 Department of Mathematics, University of Jhang, Jhang, 35200 Pakistan; 5 Department of Mathematics, Lahore College for Women University, Lahore, 54000 Pakistan; 6 Department of Mathematics, College of Arts and Sciences, Prince Sattam Bin Abdulaziz University, Wadi Aldawaser, 11991 Saudi Arabia; 7 Department of Mathematics, Faculty of Science, New Valley University, Al-Kharga, Al-Wadi Al-Gadid, 72511 Egypt; 8 Department of Mathematics, Faculty of Science, Northern Border University, Arar, 1321 Saudi Arabia; 9 Department of Physics, College of Sciences, University of Bisha, P.O. Box 344, Bisha, 61922 Saudi Arabia; 10 Physics Department, Faculty of Science, Al-Azhar University, Assiut, 71524 Egypt; 11 Advanced Functional Materials and Optoelectronic Laboratory (AFMOL), Department of Physics, Faculty of Science, King Khalid University, P.O. Box 9004, Abha, Saudi Arabia; 12 Research Center for Advanced Materials Science (RCAMS), King Khalid University, P.O. Box 9004, Abha, 61413 Saudi Arabia; 13 Nanoscience Laboratory for Environmental and Biomedical Applications (NLEBA), Semiconductor Lab., Department of Physics, Faculty of Education, Ain Shams University, Roxy, Cairo, 11757 Egypt

**Keywords:** Mathematics and computing, Physics

## Abstract

Nowadays, with the advantages of nanotechnology and solar radiation, the research of Solar Water Pump (SWP) production has become a trend. In this article, Prandtl–Eyring hybrid nanofluid (P-EHNF) is chosen as a working fluid in the SWP model for the production of SWP in a parabolic trough surface collector (PTSC) is investigated for the case of numerous viscous dissipation, heat radiations, heat source, and the entropy generation analysis. By using a well-established numerical scheme the group of equations in terms of energy and momentum have been handled that is called the Keller-box method. The velocity, temperature, and shear stress are briefly explained and displayed in tables and figures. Nusselt number and surface drag coefficient are also being taken into reflection for illustrating the numerical results. The first finding is the improvement in SWP production is generated by amplification in thermal radiation and thermal conductivity variables. A single nanofluid and hybrid nanofluid is very crucial to provide us the efficient heat energy sources. Further, the thermal efficiency of MoS_2_–Cu/EO than Cu–EO is between 3.3 and 4.4% The second finding is the addition of entropy is due to the increasing level of radiative flow, nanoparticles size, and Prandtl–Eyring variable.

## Introduction

Green electric energy is attained from the heat radiated and converted light from the sun. Aside from the fact that solar energy is renewable, it requires minimal maintenance and it can also generate electricity and heat, which may cause a depletion in power costs. We have two primary forms of solar energy: photovoltaic (PV) and concentrated solar power (CSP). In the PV solar panels, sun rays are stored by solar cells, which are then transformed directly into energy. The primary application of PV systems, combined with the solar battery, are (i) public lighting (billboards, highway, parking lots)^1^, (ii) amplification of signals in communication systems (wireless)^2^, and (iii) solar water pump^3^.

Meanwhile, CSP systems use mirrors (lenses and reflectors) to collect sun rays to heat a liquid substance. The high temperature of the liquid in CSP causes a heat engine to work. The main applications of CSP are (i) water heating for hostel^4^, (ii) greenhouse plants^5^, and (iii) biogas production^6^. Furthermore, CSP systems are effective in storing energy using thermal energy storage technologies (TES). Therefore, CSP systems can be employed for no or low sunlight, e.g., on the weathers of clouds or in the nighttime, to produce electric power. This methodology enhances the usage of the technology of solar thermal conductivity as it can deal with environmental limitations. CSP systems are the most fascinating due to high power generation because they utilized TES. This type of thermal storage (TES) is considered more efficient in a solar battery because it can control energy supply and demand. Besides, the utilization of TES reduces the following factors: energy demand, energy consumption, and cost. In a conclusion, the efficiency of CSP systems can be enhanced^7^. Advantages of combining storage and solar battery are balancing electricity loads, stability solar generation, and providing resilience.

Solar Water Pumps (SWP) is one of the applications from PV solar systems, which produces electricity to pump water. Typical applications are water for the agricultural sector^8^, crop irrigation^9^, and livestock^10^. In 1901, A. Eneas built the giant solar water pump globally at the Ostrich Farm in Pasadena, California, USA^11^. Subsequently, in 1920, Harrington from New Mexico was the first one who used the solar concentrating technology [also known as concentrated solar power (CSP)], in the form of the solar-powered steam engine to pump water up to a height of 6 m^11^. In conclusion, the literature review proved that SWP applied both solar technology: PV and CSP, to perform its works.

One type of CSP is parabolic trough solar collector (PTSC), which has four fundamental elements: collector, receiver, heat transfer fluid (HTF), and heat engine. A collector is a reflecting surface that uses a parabolic mirror to reflect the collective thermal energy from the sun to the receiver. This receiver transfers the heat to the focal line, which is filled with an HTF. The temperature of HTF will become very high (almost 390 °C), and it will flow to the heat engine to generate electricity. The heat transfer in HTF is a cyclic process, where the process is continued in the power generation systems. The ideal characteristics of HTF in PTSC are: can work at high temperature, stable at high temperature, minimum viscosity, minimum vapor pressure, the minimum point of freezing, low maintenance including costs, non-corrosive, and proven as safe^12^. Lately, it is found that nanofluids are particular HTF with advanced thermal and optical characteristics. The most usual nanoparticles in a nanofluid as HTF in PTSC are metallic and non-metallic nanoparticles. Some of the examples of metallic nanoparticles in nanofluid as HTC are Al^13^, Au^14^, Ag, and Cu^15^. Meanwhile, non-metallic nanoparticles consisting of the examples of ZnO^14^, CeO_2_^16^, NiFe_2_O_4_^17^, Fe_2_O_3_^18^, Fe_3_O_4_^19^, SiO_2_, CuO, TiO_2_, and Al_2_O_3_^20^. Other types of particles in nanofluid, such as carbon nanotubes (CNT)^21^, single-walled carbon nanotubes (SWCNT)^22^, and multi-walled carbon nanotubes (MWCNT)^23^ also being applied in the experimental and numerical works in PTSC.

Hybrid nanofluid has become one of the selections as HTF in PTSC due to its higher thermal conductivity^24^ than nanofluid. It is prepared by dispersing two or more different nanoparticles in the base fluid. Studies using hybrid nanofluids in PTSC have been reported, by considering the following nanoparticles: Ag–MgO/water^13^, GO–Co_3_O_4_/60EG:40 W^13^, Cu–Al_2_O_3_/water^13^, Al_2_O_3_–TiO_2_/Syltherm 800^25^, Ag–ZnO/Syltherm 800, Ag–TiO_2_/Syltherm 800, and Ag–MgO/Syltherm 800^26^.

The entropy of an isolated system is continuously increasing that is the second law of thermodynamics. Therefore, some studies have described this law in thermal system performance^27^ and heat transfer in nanofluid^28^. Moreover, entropy generation causes a decline in the thermal system^29^. So, the researchers must find a way to control the entropy level to increase the thermal conduction in flowing fluid. One of the solutions is to include entropy in their fluid flow model. The recent studies of entropy in hybrid nanofluid have been reported^30–37^. The magnetohydrodynamics (MHD) flow of water based hybrid nanofluid was examined by Khan et al.^30^ and Shah et al.^31^. In contrast, the MHD hybrid nanofluid where the base fluid is Ethylene glycol was described by Aziz et al.^32^. The effect of thermal radiation was studied by Jamshed and Aziz^33^ and Aziz et al.^34^, where ethylene glycol^33^ and water^34^ were acting as a base for hybrid nanofluid. In addition, Jamshed and Aziz^33^ studied the Casson hybrid nanofluid, whereas Aziz et al.^34^ analyzed the Powell–Eyring hybrid nanofluid. The thermal characteristics of hybrid nanofluid in the various shape of container or boundary, such as in microchannel^35^, rotating channel^36^, and wavy cavity^37^, were reported. These studies were emphasizing the model of Cu–Ti and C71500 water-base fluid^35^, Cu–Al_2_O_3_ ethylene glycol base fluid^36^, and Cu–Al_2_O_3_ water-base fluid^37^.

The Prandtl–Eyring fluid (PEF) is a type of non-Newtonian viscoelastic fluid ideal talented of labeling nil shear degree viscosity possessions which widening of a mass tempts the flow. The scientific preparation of the issue provides an exceedingly set of nonlinear partial differential equations. Arif et al.^38^ establish a theoretical review to detect the impacts of a customarily employed magnetic field on PEF flow through a linearly stretchable surface. Abbas et al.^39^ studied a three-dimensional axisymmetric inactivity flow using PEF. Khan et al.^40^ proposed a mathematical analysis of bio convection on PEF, whereas Abdelmalek et al.^41^ investigated the influences of Brownian motion and thermo/phonetic stress on a thermal exchange of PFF produced by strained shallow. Abbasi et al.^42^ studied the effects on electro-osmosis-modulated peristaltic flow of PEF through the pointed strait. Finally, Yong et al.^43^ and Imran et al.^44^ presented a mathematical modeling system having the PEF flow. Recent additions considering nanofluids with heat and mass exchange in different physical aspects are given by^33, 34, 45–58^.

**Figure 1:**
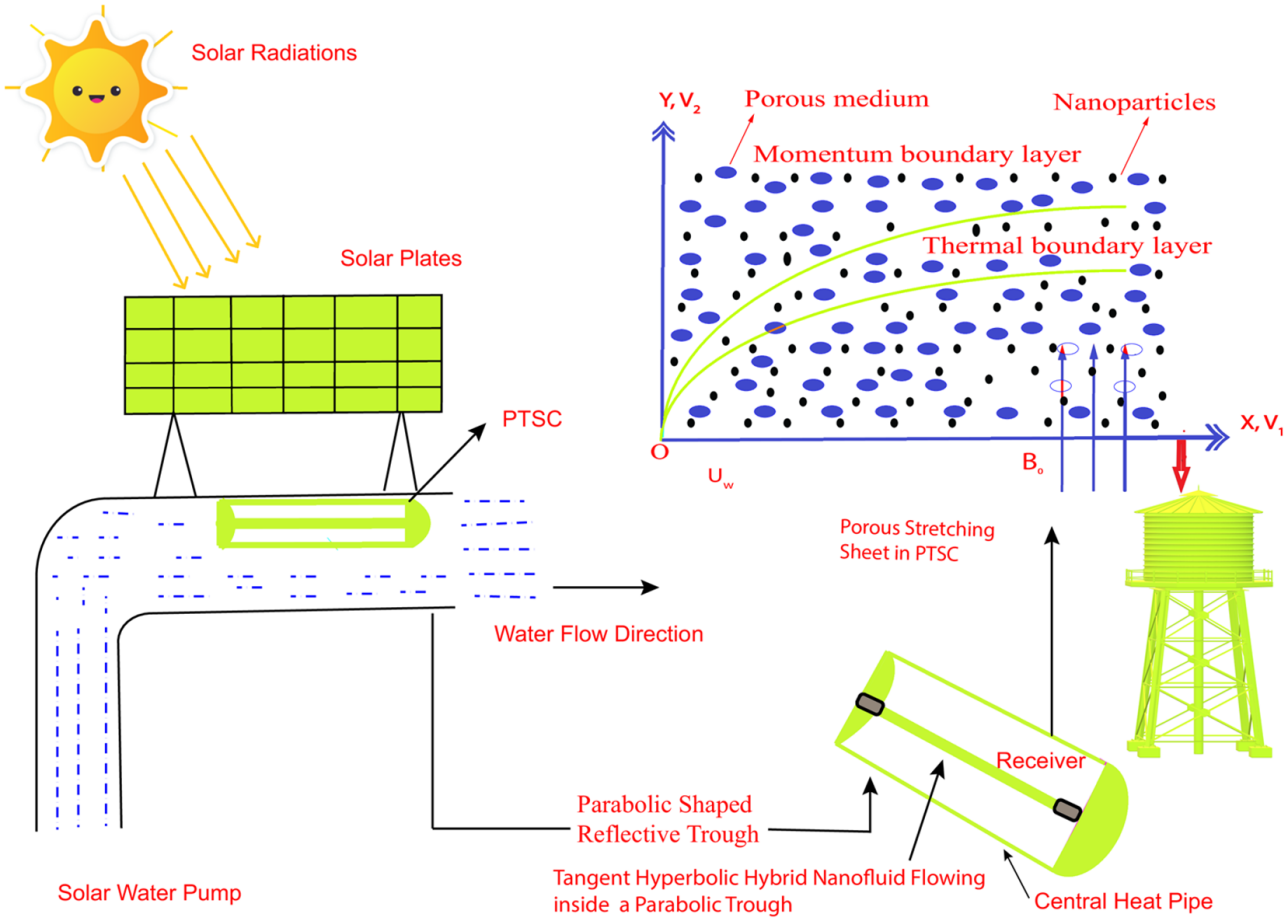
The theoretical experiment of a solar water pump.

Based on the debates above, the main objective of the present exploration focuses on the influences of viscous dissipation, heat radiations, heat source, and entropy generation analysis on Prandtl–Eyring hybrid nanofluid flow (P-EHNF) which is chosen as a working fluid in the production of SWP in PTSC is investigated. Cu and MoS_2_ nanoparticles in EO as a base fluid are considered. The comparing of thermal features between the single (Cu–EO) and hybrid (MoS_2_–Cu/EO) nanofluid are demonstrated. By using a well-established numerical scheme the group of equations in terms of energy and momentum have been handled that is called the Keller-box method. The velocity, temperature, and shear stress are briefly explained and displayed in tables and figures. Nusselt number and surface drag coefficient are also being taken into reflection for illustrating the numerical results. Figure 1 represents a parabolic trough solar collector in a water pump.

### Novelty of current work

A mathematical formulation of solar water pumps using Prandtl–Eyring and PTSC hybrid nanofluid was originated accordingly in the earlier studies, which were explained in the previous sections. Molybdenum disulfide oxide (MoS_2_) and Copper (Cu) are the types of nanoparticles in this fluid because engine oil is the base fluid (EO). This article's crucial contribution includes the following: there has been no actual achievement of this initial theoretical experiment. The third-world or undeveloped nations can use this PTSC as a radical source of energy by exploiting the light and heat from the sun. Our analysis uses PTSC, which needs the selection of a suitable working fluid. In a conclusion, hybrid nanofluid has replaced nanofluid as our first option due to the variety that it has a greater rate of heat transmission.

### Outline of current work

Our paper has been structured as:

- In section “Flow model formulations”, we gave a regarding mathematical formulated.
- In section “Dimensionless formulations model”, we have developed the solution to the problem.
- The numbering process was provided in section “Classical Keller-box technique”, the Keller-box system.
- Then in section “Code verification”, we developed code validation.
- In section “Entropy analysis”, we analyzed the development of entropy.
- In section “Results and discussion”, we summarized the findings and the debate.
- Finally, in section “Final results and future guidance”, we have achieved the final results and futuristic guidelines.

## Flow model formulations

The equations of the mathematical flow model illustrated a horizontally moving plate through an uneven expansion velocity ${U}_{w}\left(x,0\right)$, given that *b* is the unique expansion rate such as:

1 $${U}_{w}\left(x,0\right)=bx.$$

The remote exterior temperature is denoted by ${{\yen }}_{w}(x,t)={{\yen }}_{\infty }+{b}^{*}x$ whereas it supposedly will become constant when $x=0$. The notation ${{\yen }}_{\infty },{{\yen }}_{w}$ and ${b}^{*}$ gave out the meaning of temperature of neighboring, exterior as well as variation rate, congruently. Besides being slippery, the plate's surface is sensitive to temperature fluctuations. At an interaction volume fraction (${\phi }_{Cu}$), a fixed value of 0.09 Cu nano solid-particles are added to the EO-based fluid to produce a hybrid nanofluid for this study. A hybrid nanofluid composed of molybdenum disulfide (MoS_2_) nano molecules have been created at a concentrated size ${\phi }_{Ms}$.

### Suppositions and terms of model

The principles and constraints apply to the flow model can be described as follows:

- 2-D laminar flowing.
- Boundary-layer approximations.
- Single phase (Tiwari-Das) scheme.
- Solar water pump.
- Parabolic trough solar collector.
- Non-Newtonian P-EHNF.
- Porous media.
- Thermal radiative flow.
- Nano solid-particles shape-factor.
- Porousnesselongated surface.
- Convection and slippery boundary constraints.

### Geometric model

**Figure 2:**
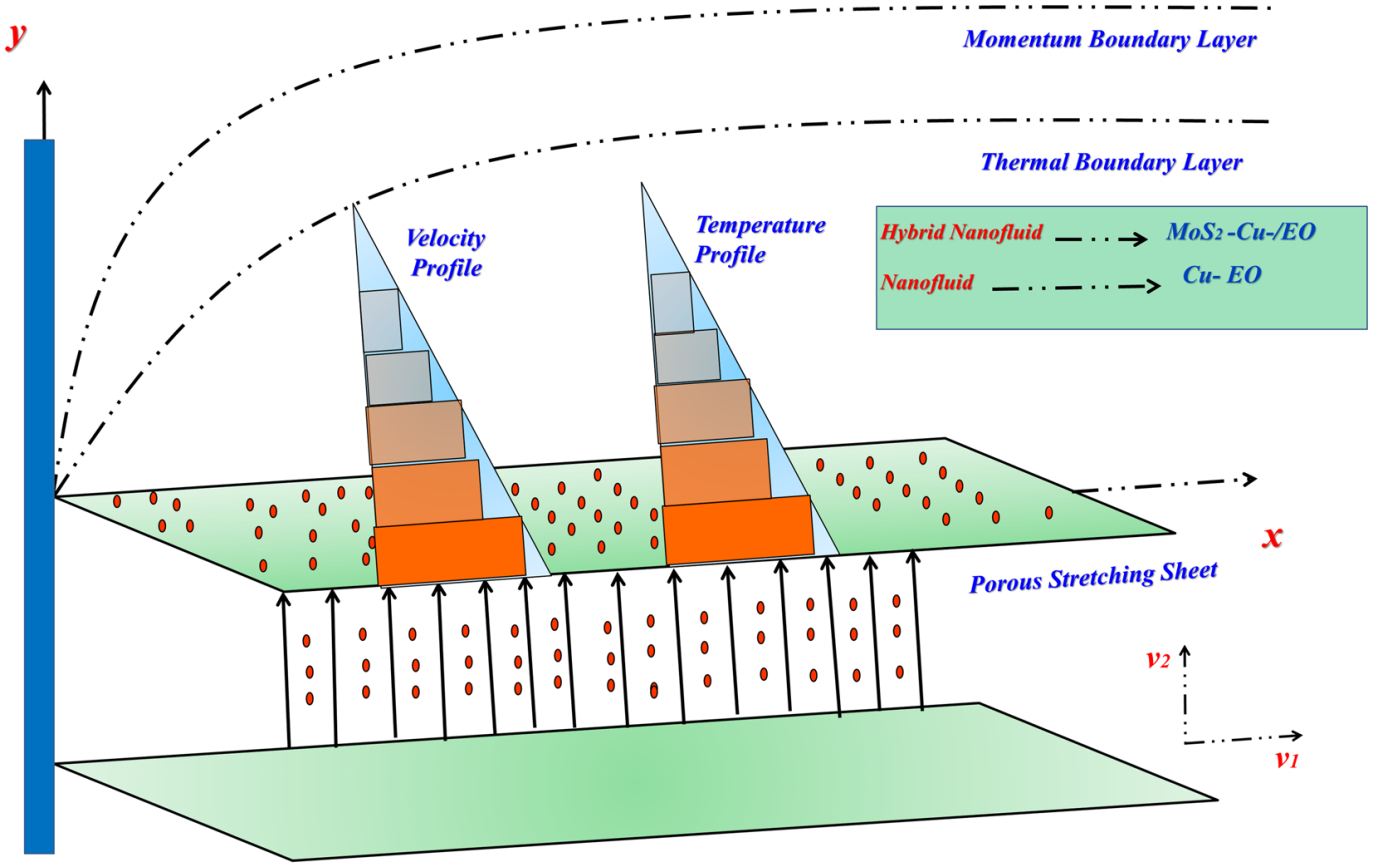
Diagram of the flow model.

The geometric flowing model is displayed as in Fig. 2:

### Model equations

The constitutive flow formulas^45^ of the viscous Prandtl–Eyring hybrid nanofluid, in combination with a porous media, viscous dissipation, and thermal radiative flow utilizing the approximate boundary layer, are

2 $$\frac{\partial {v}_{1}}{\partial x}+\frac{\partial {v}_{2}}{\partial y}=0,$$

3 $${v}_{1}\frac{\partial {v}_{1}}{\partial x}+{v}_{2}\frac{\partial {v}_{1}}{\partial y}=\frac{{A}_{\mathrm{d}}}{C{\rho }_{hnf}}\left(\frac{{\partial }^{2}{v}_{1}}{\partial {y}^{2}}\right)-\frac{{A}_{\mathrm{d}}}{{2{C}^{3}\rho }_{hnf}}\frac{{\partial }^{2}{v}_{1}}{\partial {y}^{2}}\left[{\left(\frac{\partial {v}_{1}}{\partial y}\right)}^{2}\right]-\frac{{\mu }_{hnf}}{{\rho }_{hnf}k}{v}_{1},$$

4 $${v}_{1}\frac{\partial {\yen }}{\partial x}+{v}_{2}\frac{\partial {\yen }}{\partial y}=\frac{{k}_{hnf}}{(\rho {C}_{p}{)}_{hnf}}\left(\frac{{\partial }^{2}T}{\partial {y}^{2}}\right)-\frac{1}{{\left(\rho {C}_{p}\right)}_{hnf}}\left(\frac{\partial {q}_{r}}{\partial y}\right)+\frac{1}{{\left(\rho {C}_{p}\right)}_{hnf}}{Q}_{L}\left(T-{T}_{\infty }\right)+\frac{{\mu }_{hnf}}{{\left(\rho {C}_{p}\right)}_{hnf}}{\left(\frac{\partial {v}_{1}}{\partial y}\right)}^{2}.$$

The related boundary constraints are Aziz et al.^46^:

5 $${v}_{1}(x,0)={U}_{w}+{N }_{L}\left(\frac{\partial {v}_{1}}{\partial y}\right),{v}_{2}(x,0)={V}_{w}, \quad -{k}_{\mathrm{L}}\left(\frac{\partial {\yen }}{\partial y}\right)={h}_{L}({{\yen }}_{w}-{\yen }),$$

6 $${v}_{1}\to 0, \quad {\yen }\to {{\yen }}_{\infty } as y\to \infty .$$

where the component of flowing velocity can be prescribed as $\overleftarrow{v}=[{v}_{1}(x,y,0),{v}_{2}(x,y,0),0]$. We consider ${\yen }$ as the temperature of the fluid. Other vital quantities are fluid parameters ${A}_{\mathrm{d}}$ and ${\mathrm{C}},$ surface permeability ${V}_{w}$, heat transfer coefficient ${h}_{L}$, porosity $(k)$ and thermal conductivity of solid ${k}_{\mathrm{L}}$. Physical behaviours comprising convectional heated surface approaches to its thermal wastage by the application of conduction (Newtonian heating), and flow velocity in the neighboring of the surface has a direct behaviour to the shear stress exerts in it (slip condition) are taken into account.

### Thermo-physical properties of P-ENF

Nano solid particles dispersed in EO induce improved thermophysical characteristics. The following Table 1 equations summarize P-ENF substance variables^47, 48^.

**Table 1:** Thermo-physical features for nanoliquids.

Features	Nanoliquid
Dynamical viscidness $\left( \mu \right)$	$\mu_{nf} = \mu_{f} (1 - \phi )^{ - 2.5}$
Density $\left( \rho \right)$	$\rho_{nf} = \left( {1 - \phi } \right)\rho_{f} + \phi \rho_{s}$
Heat capacity $\left( {\rho C_{p} } \right)$	$(\rho C_{p} )_{nf} = \left( {1 - \phi } \right)(\rho C_{p} )_{f} + \phi (\rho C_{p} )_{s}$
Thermal conductivity $\left( \kappa \right)$	$\frac{{\kappa_{nf} }}{{\kappa_{f} }} = \left[ {\frac{{\left( {\kappa_{s} + 2\kappa_{f} } \right) - 2\phi \left( {\kappa_{f} - \kappa_{s} } \right)}}{{\left( {\kappa_{s} + 2\kappa_{f} } \right) + \phi \left( {\kappa_{f} - \kappa_{s} } \right)}}} \right]$

$\phi$ is the nano solid-particle size coefficient. $\mu_{f}$, $\rho_{f}$, $(C_{p} )_{f}$ and $\kappa_{f}$ are dynamical viscidness, intensity, operative heat capabilities, and thermal exchange of the standard fluid respectively. The useless characteristics $\rho_{s}$, $(C_{p} )_{s}$ and $\kappa_{s}$ are the intensity, effective heat capacitance, and thermal conductance of the nanomolecules, correspondingly

### Thermo-physical properties of P-EHNF

The primary assumption of hybrid nanofluids is the insertion of two distinct nanosolid particles inside the basis fluid^49^. Theseacts improve the capacity for heat transmission of common liquids and are a maximum heat interpreter under nanofluids. P-EHNF variables content is summarized in Table 2^33, 34^.

**Table 2:** Thermo-physical properties of hybrid nanofluids.

Features	Hybrid nanofluid
Viscosity $\left( \mu \right)$	$\mu_{hnf} = \mu_{f} (1 - \phi_{Cu} )^{ - 2.5} (1 - \phi_{Ms} )^{ - 2.5}$
Density $\left( \rho \right)$	$\rho_{hnf} = \left[ {\left( {1 - \phi_{Ms} } \right)\left\{ {\left( {1 - \phi_{Cu} } \right)\rho_{f} + \phi_{Cu} \rho_{{p_{1} }} } \right\}} \right]$ + $\phi_{Ms} \rho_{{p_{2} }}$
Heat capacity $\left( {\rho C_{p} } \right)$	$(\rho C_{p} )_{hnf} = [\left( {1 - \phi_{Ms} } \right)\{ \left( {1 - \phi_{Cu} } \right)(\rho C_{p} )_{f} + \phi_{Cu} (\rho C_{p} )_{{p_{1} }} \} ] + \phi_{Ms} (\rho C_{p} )_{{p_{2} }}$
Thermal conductivity $\left( \kappa \right)$	$\frac{{\kappa_{hnf} }}{{\kappa_{gf} }} = \left[ {\frac{{\left( {\kappa_{{p_{2} }} + 2\kappa_{gf} } \right) - 2\phi_{Ms} \left( {\kappa_{gf} - \kappa_{{p_{2} }} } \right)}}{{\left( {\kappa_{{p_{2} }} + 2\kappa_{gf} } \right) + \phi_{Ms} \left( {\kappa_{gf} - \kappa_{{p_{2} }} } \right)}}} \right]$; $\frac{{\kappa_{gf} }}{{\kappa_{f} }} = \left[ {\frac{{\left( {\kappa_{{p_{1} }} + 2\kappa_{f} } \right) - 2\phi_{Cu} \left( {\kappa_{f} - \kappa_{{p_{1} }} } \right)}}{{\left( {\kappa_{{p_{1} }} + 2\kappa_{f} } \right) + \phi_{Cu} \left( {\kappa_{f} - \kappa_{{p_{1} }} } \right)}}} \right]$

In Table 2, ${\mu }_{hnf}$, ${\rho }_{hnf}$, $\rho ({C}_{p}{)}_{hnf}$ and ${\kappa }_{hnf}$ are hybrid nanofluid dynamical viscidness, intensity, consistent heat capability, and thermal conduction. $\phi$ is the volume of solid nanomolecules coefficient for mono nanofluid and ${\phi }_{hnf}={\phi }_{Cu}+{\phi }_{Ms}$ is the nano soli-particles size coefficient for the mixture of nanofluid. ${\mu }_{f}$, ${\rho }_{f}$, $({C}_{p}{)}_{f}$, ${\kappa }_{f}$ and ${\sigma }_{f}$ are dynamical viscidness, density, specific heat capacitance, and thermal conduction of the basefluid. ${\rho }_{{p}_{1}}$, ${\rho }_{{p}_{2}}$, $({C}_{p}{)}_{{p}_{1}}$, $({C}_{p}{)}_{{p}_{2}}$, ${\kappa }_{{p}_{1}}$ and ${\kappa }_{{p}_{2}}$ are the intensity, specific heat capacitance, and thermal conductance of the nanomolecules.

### Nano solid-particles and basefluid lineaments

In this analysis, the material characteristics of the primary engine oil-based liquid of the engine are specified in Table 3^50, 51^.

**Table 3:** Fabricated materials thermo-physical attributes.

Thermophysical	$\rho \;({\text{kg/m}}^{3} )$	$c_{p} \;({\text{J/kg}}\;{\text{K}})$	$k\;({\text{W/mK}})$
Copper (Cu)	8933	385	401
Engine oil (EO)	884	1910	0.144
Molybdenum disulfide (MoS_2_)	5060	397.746	34.5
(Cu–EO) P-ENF	2332.82	765,464	0.30573
(MoS_2_–Cu/EO) P-EHNF	2823.71	2,005,201	0.63584

### Rosseland approximation

Radiative flow only passes a shortened distance because its non-Newtonian P-EHNF is thicker. Because of this, the approximation for radiative fluxing from Rosseland^52^ is utilized in the formula (4).

7 $${q}_{r}=-\frac{4{\sigma }^{*}}{3{k}^{*}}\frac{\partial {T}^{4}}{\partial y},$$

herein, ${\sigma }^{*}$ signifies the constant worth of Stefan-Boltzmann and ${k}^{*}$ symbols the rate.

## Dimensionless formulations model

In the study of the similarity technology that transmutes the governing PDEs into ODEs, the BVP formulas (2)–(6) are modified. Familiarizing stream function $\psi$ in the formula

8 $${v}_{1}=\frac{\partial \psi }{\partial y},{v}_{2}=-\frac{\partial \psi }{\partial x}.$$

The specified similarity quantities are

9 $$\Gamma (x,y)=\sqrt{\frac{b}{{\nu }_{f}}}y, \quad \psi (x,y)=\sqrt{{\nu }_{f}b}xf(\chi ), \quad \theta (\chi )=\frac{{\yen }-{{\yen }}_{\infty }}{{{\yen }}_{w}-{{\yen }}_{\infty }}.$$

into Eqs. (2)–(6). We get

10 $${\alpha }^{*}f{^{\prime\prime\prime}}(1-{\beta }^{*}{f{^{\prime}}{^{\prime}}}^{2})+{\phi }_{b}\left[ff{^{\prime}}{^{\prime}}-{f{^{\prime}}}^{2}\right]-\frac{1}{{\phi }_{a}}Kf{^{\prime}}=0,$$

11 $$\theta {^{\prime\prime}}\left(1+\frac{1}{{\phi }_{d}}{P}_{r}{N}_{L}\right)+{P}_{r}\frac{{\phi }_{c}}{{\phi }_{d}}\left[f\theta {^{\prime}}-f{^{\prime}}\theta +\frac{{E}_{L}}{{\phi }_{a}{\phi }_{c}}{f{^{\prime}}{^{\prime}}}^{2}\right]=0.$$

with

12 $$\left.\begin{array}{l}f(0)=S, f{^{\prime}}(0)=1+{\Lambda }_{L}f{^{\prime \prime}}(0), \quad \theta {^{\prime}}(0)=-{G}_{L}(1-\theta (0))\\ f{^{\prime}}(\Gamma )\to 0, \theta (\Gamma )\to 0, as\Gamma \to \infty \end{array}\right\}$$

where ${\phi {^{\prime}}}_{i}s$ is $a\le i\le d$ in formulas (10) and (11) signify the subsequent thermo-physical structures for P-HNF

13 $${\phi }_{a}=(1-{\phi }_{Cu}{)}^{2.5}(1-{\phi }_{Ms}{)}^{2.5},{\phi }_{b}=(1-{\phi }_{Ms})\left[(1-{\phi }_{Cu})+{\phi }_{Cu}\frac{{\rho }_{{p}_{1}}}{{\rho }_{f}}\right]+{\phi }_{Ms}\frac{{\rho }_{{p}_{2}}}{{\rho }_{f}},$$

14 $${\phi }_{c}=(1-{\phi }_{Ms})\{(1-{\phi }_{Cu})+{\phi }_{Cu}\frac{(\rho {C}_{p}{)}_{{p}_{1}}}{(\rho {C}_{p}{)}_{f}}\}+{\phi }_{Ms}\frac{(\rho {C}_{p}{)}_{{p}_{2}}}{(\rho {C}_{p}{)}_{f}},$$

15 $${\phi }_{d}=\left[\frac{({\kappa }_{{p}_{2}}+2{\kappa }_{nf})-2{\phi }_{Ms}({\kappa }_{nf}-{\kappa }_{{p}_{2}})}{({\kappa }_{{p}_{2}}+2{\kappa }_{nf})+{\phi }_{Ms}({\kappa }_{nf}-{\kappa }_{{p}_{2}})}\right]\left[\frac{({\kappa }_{{p}_{1}}+2{\kappa }_{f})+{\phi }_{Cu}({\kappa }_{f}-{\kappa }_{{p}_{1}})}{({\kappa }_{{p}_{1}}+2{\kappa }_{f})-2{\phi }_{Cu}({\kappa }_{f}-{\kappa }_{{p}_{1}})}\right].$$

### Description of the embedded control physical parameters

Equation (2) is precisely approved. In already discussed equations, the notation ${^{\prime}}$ was employed for expressing the derivatives related to $\Gamma$. Here

**Table:** 

Parameter	Name	Expression	Default value
${\alpha }^{*}$	Prandtl–Eyring parameter-I	${\alpha }^{*}=\frac{{A}_{\mathrm{d}}}{{\mu }_{fC}}$	1.0
${\beta }^{*}$	Prandtl–Eyring parameter-II	${\beta }^{*}=\frac{{b}^{*}{x}^{2}}{2{C}^{2}{\nu }_{f}}$	0.4
${P}_{r}$	Prandtl number	${P}_{r}$ =$\frac{{\nu }_{f}}{{\alpha }_{f}}$	6450
$\phi$	Volume fraction	$\phi$	0.18
*K*	Porosity parameter	$K=\frac{{\nu }_{f}}{bk}$	0.1
$S$	Suction/injection parameter	$S=-{V}_{w}\sqrt{\frac{1}{{\nu }_{f}b}}$	0.4
${N}_{r}$	Thermal radiation parameter	${N}_{L}=\frac{16}{3}\frac{{\sigma }^{*}{{\yen }}_{\infty }^{3}}{{\kappa }^{*}{\nu }_{f}(\rho {C}_{p}{)}_{f}}$	0.3
*G*_*L*_	Biot number	${G}_{L}=\frac{{h}_{L}}{\mathrm{L}}\sqrt{\frac{{\nu }_{f}}{b}}$	0.3
*E*_*L*_	Eckert number	${E}_{L}=\frac{{U}_{w}^{2}}{({C}_{p}{)}_{f}({{\yen }}_{w}-{{\yen }}_{\infty })}$	0.3
${\Lambda }_{L}$	Velocity slip	${\Lambda }_{L}=\sqrt{\frac{b}{{\nu }_{f}}}{\mu }_{f}$	0.3

### Drag force and Nusselt number

The drag force $({C}_{f})$ combined with the Nusselt amount $(N{u}_{x})$ are the interesting physical amounts that controlled the flowing and specified as^45^

16 $${C}_{f}=\frac{{\tau }_{w}}{\frac{1}{2}{\rho }_{f}{U}_{w}^{2}}, \quad N{u}_{x}=\frac{x{q}_{w}}{{k}_{f}({{\yen }}_{w}-{{\yen }}_{\infty })}$$

where ${\tau }_{w}$ and ${q}_{w}$ determine as

17 $${\tau }_{w}={\left(\frac{{A}_{\mathrm{d}}}{C}\frac{\partial {v}_{1}}{\partial y}+\frac{{A}_{\mathrm{d}}}{6{C}^{3}}{\left(\frac{\partial {v}_{1}}{\partial y}\right)}^{3}\right)}_{y=0},{q}_{w}=-{k}_{hnf}\left(1+\frac{16}{3}\frac{{\sigma }^{*}{{\yen }}_{\infty }^{3}}{{\kappa }^{*}{\nu }_{f}(\rho {C}_{p}{)}_{f}}\right){\left(\frac{\partial {\yen }}{\partial y}\right)}_{y=0}$$

The dimensionless transmutations (9) are implemented to obtain

18 $${C}_{f}R{e}_{x}^\frac{1}{2}={\alpha }^{*}{f}^{{^{\prime \prime}}}\left(0\right)-\frac{1}{3}{\alpha }^{*}{\beta }^{*}{\left(f{^{\prime \prime}}(0)\right)}^{3}, \quad N{u}_{x}R{e}_{x}^{-\frac{1}{2}}=-\frac{{k}_{hnf}}{{k}_{f}}\left(1+{N}_{L}\right)\chi {^{\prime}}(0),$$

where $N{u}_{x}$ increases the amount of Nusselt and ${C}_{f}$ causes an increment in the drag force coefficient. $R{e}_{x}=\frac{{u}_{w}x}{{\nu }_{f}}$ is local $Re$ based on the elongated velocity ${u}_{w}(x)$.

## Classical Keller-box technique

**Figure 3:**
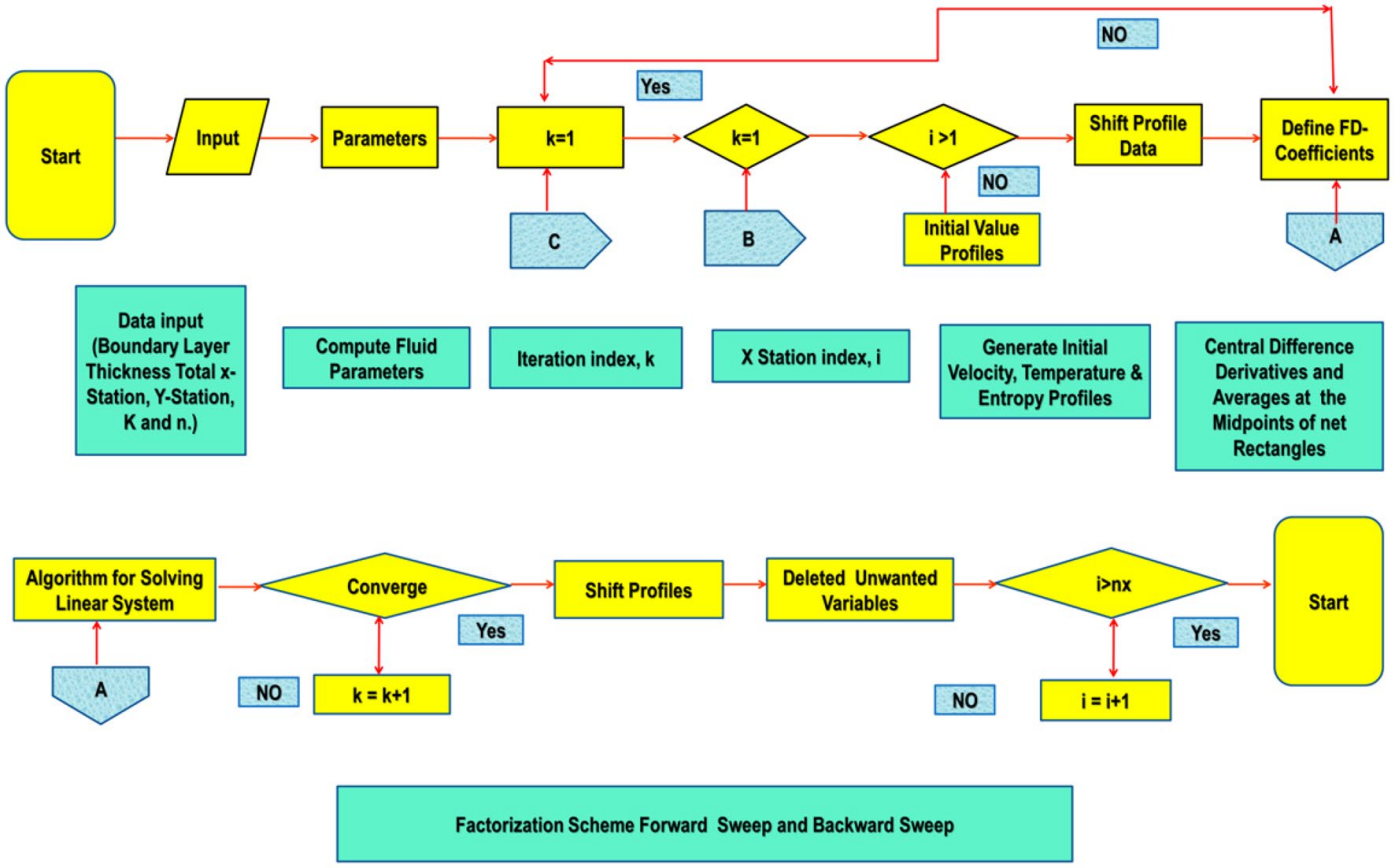
Chart of KBM steps.

Because of its rapid convergence, the Keller-box approach (KBM)^53^ is used to find solutions for model formulas (Fig. 3). KBM is used to find the localized solve of (10) and (11) with constraints (12). The policy of KBM is specified as next.

### Stage 1: ODEs adaptation

In the early stage, all of the ODEs must be transformed into 1st-order ODEs (10)–(12)

19 $${z}_{1}={f}^{^{\prime}},$$

20 $${z}_{2}={z}_{1}^{^{\prime}},$$

21 $${z}_{3}={\theta }^{^{\prime}},$$

22 $${\alpha }^{*}{z}_{2}^{^{\prime}}(1-{\beta }^{*}{{z}_{2}}^{2})+{\phi }_{b}\left[f{z}_{2}-{z}_{1}^{2}\right]-\frac{1}{{\phi }_{a}}K{z}_{1}=0,$$

23 $${z}_{3}^{^{\prime}}\left(1+\frac{1}{{\phi }_{d}}{P}_{r}{N}_{r}\right)+{P}_{r}\frac{{\phi }_{c}}{{\phi }_{d}}\left[f{z}_{3}-{z}_{1}\theta +\frac{{E}_{L}}{{\phi }_{a}{\phi }_{c}}{{z}_{2}}^{2}\right]=0.$$

24 $$f(0)=S,{z}_{1}(0)=1+{\Lambda }_{L}{z}_{2}(0),{z}_{3}(0)=-{G}_{i}(1-\theta (0)),{z}_{1}(\infty )\to 0,\theta (\infty )\to 0.$$

### Stage 2: domain discretization

**Figure 4:**
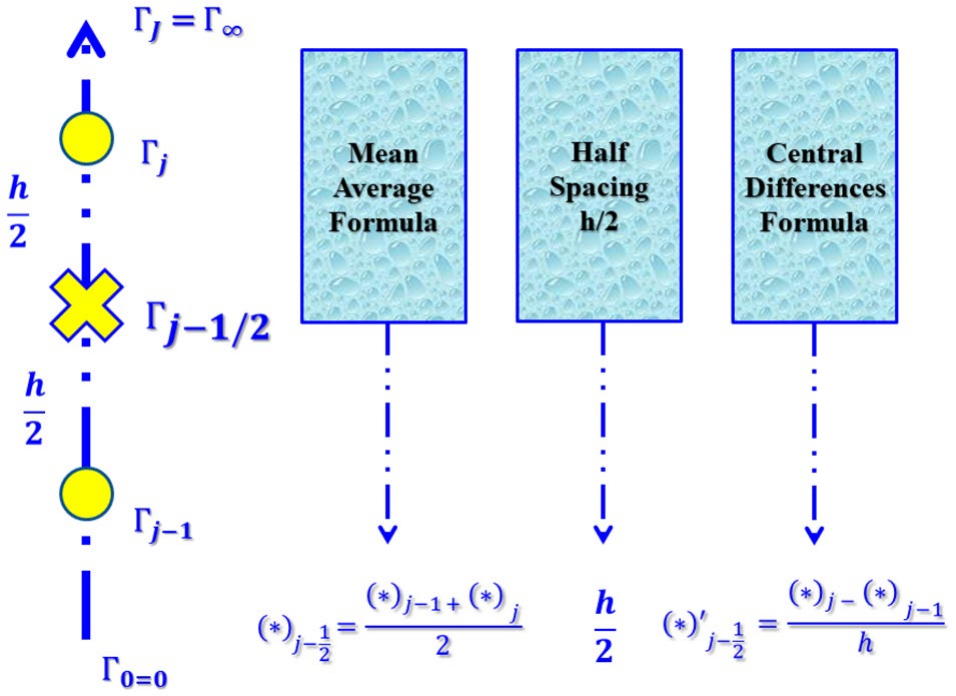
The rectangular grid of difference approximation.

The process domain has to be discretized to compute the estimated solution. Usually, the field is separated into the same grid size by discretization (Fig. 4). The computational findings gain a minor grid relatively with high precision. ${\Gamma }_{0}=0, {\Gamma }_{j}={\Gamma }_{j-1}+h, j=1,2,3,\ldots,J-1, {\Gamma }_{J}={\Gamma }_{\infty }$. The $j$ is used for providing a $h$-spacing in a horizontal direction to show the position of the coordinates. The solution is calculated without the use of any initial approximation, so it is essential to measure speed, temperatures, entropy changes, and temperature variations, to make a basic estimation among $\Gamma =0$ and $\Gamma =\infty$. The obtained designs are approximated solutions provided that they fulfill the criteria of boundary constraints of the problem. It is significant to observe here that the outputs would be equal in the end when different basic estimations are being chosen, but that the iteration count and time are considered to transfer the calculations, which may change (Fig. 3).

Differences formulas are calculated using center differences, and average functions are replaced. The 1st ODEs (19)–(23) order is then reduced to the next series of non-linear algebraic equations.

25 $$\frac{({z}_{1}{)}_{j}+({z}_{1}{)}_{j-1}}{2}=\frac{{f}_{j}-{f}_{j-1}}{h},$$

26 $$\frac{({z}_{2}{)}_{j}+({z}_{2}{)}_{j-1}}{2}=\frac{({z}_{1}{)}_{j}-({z}_{1}{)}_{j-1}}{h},$$

27 $$\frac{({z}_{3}{)}_{j}+({z}_{3}{)}_{j-1}}{2}=\frac{{\theta }_{j}-{\theta }_{j-1}}{h},$$

28 $$\begin{array}{l}{\alpha }^{*}\left(\frac{({z}_{2}{)}_{j}-({z}_{2}{)}_{j-1}}{h}\right)(1-{\beta }^{*}{\left(\frac{({z}_{2}{)}_{j}+({z}_{2}{)}_{j-1}}{2}\right)}^{2})\\ +\left[{\phi }_{b}\left(\left(\frac{{f}_{j}+{f}_{j-1}}{2}\right)\left(\frac{({z}_{2}{)}_{j}+({z}_{2}{)}_{j-1}}{2}\right)-{\left(\frac{({z}_{1}{)}_{j}+({z}_{1}{)}_{j-1}}{2}\right)}^{2}\right)-K\frac{1}{{\phi }_{a}}\left(\frac{({z}_{1}{)}_{j}+({z}_{1}{)}_{j-1}}{2}\right)\right],\\ \end{array}$$

29 $$\begin{array}{l}\left(\frac{({z}_{3}{)}_{j}-({z}_{3}{)}_{j-1}}{h}\right)\left(1+\frac{1}{{\phi }_{d}}{P}_{r}{N}_{r}\right)\\ +{P}_{r}\frac{{\phi }_{c}}{{\phi }_{d}}\left[\left(\frac{{f}_{j}+{f}_{j-1}}{2}\right)\left(\frac{({z}_{3}{)}_{j}+({z}_{3}{)}_{j-1}}{2}\right)-\left(\frac{({z}_{1}{)}_{j}+({z}_{1}{)}_{j-1}}{2}\right)\left(\frac{{\theta }_{j}+{\theta }_{j-1}}{2}\right)\right]\\ \\ +{P}_{r}\frac{{\phi }_{c}}{{\phi }_{d}}\left[\frac{{E}_{L}}{{\phi }_{a}{\phi }_{c}}{\left(\frac{({z}_{2}{)}_{j}+({z}_{2}{)}_{j-1}}{2}\right)}^{2}\right]=0.\end{array}$$

### Stage 3: linearized formulas by Newton technique

The output formulas are reduced to linearity by applying the Newton technique. The ${\left(i+1\right)}{th}$ iteration can be achieved from the application of previous formulas

30 $$({)}_{j}^{(i+1)}=({)}_{j}^{(i)}+{\Theta }^{*}({)}_{j}^{(i)}.$$

We get the following linear equation method when we have the above substituted into formulas (25)–(29) and skipped the higher bounds from 2 and more of ${\Pi }_{j}^{i}$.

31 $${\Theta }^{*}{f}_{j}-{\Theta }^{*}{f}_{j-1}-\frac{1}{2}h({\Theta }^{*}({z}_{1}{)}_{j}+{\Theta }^{*}({z}_{1}{)}_{j-1})=({r}_{1}{)}_{j-\frac{1}{2}},$$

32 $${\Theta }^{*}({z}_{1}{)}_{j}-{\Theta }^{*}({z}_{1}{)}_{j-1}-\frac{1}{2}h({\Theta }^{*}({z}_{2}{)}_{j}+{\Theta }^{*}({z}_{2}{)}_{j-1})=({r}_{2}{)}_{j-\frac{1}{2}},$$

33 $${\Theta }^{*}{\theta }_{j}-{\Theta }^{*}{\theta }_{j-1}-\frac{1}{2}h({\Theta }^{*}({z}_{3}{)}_{j}+{\Theta }^{*}({z}_{3}{)}_{j-1})=({r}_{3}{)}_{j-\frac{1}{2}},$$

34 $$\begin{array}{l}({a}_{1}{)}_{j}{\Theta }^{*}{f}_{j}+({a}_{2}{)}_{j}{\Theta }^{*}{f}_{j-1}+({a}_{3}{)}_{j}{\Theta }^{*}{{z}_{1}}_{j}+({a}_{4}{)}_{j}{\Theta }^{*}{{z}_{1}}_{j-1}+({a}_{5}{)}_{j}{\Theta }^{*}{{z}_{2}}_{j}+({a}_{6}{)}_{j}{\Theta }^{*}{{z}_{2}}_{j-1}\\ +({a}_{7}{)}_{j}{\Theta }^{*}{\theta }_{j}+({a}_{8}{)}_{j}{\Theta }^{*}{\theta }_{j-1}+({a}_{9}{)}_{j}{\Theta }^{*}({z}_{3}{)}_{j}+({a}_{10}{)}_{j}{\Theta }^{*}({z}_{3}{)}_{j-1}=({r}_{4}{)}_{j-\frac{1}{2}},\end{array}$$

35 $$\begin{array}{l}({b}_{1}{)}_{j}{\Theta }^{*}{f}_{j}+({b}_{2}{)}_{j}{\Theta }^{*}{f}_{j-1}+({b}_{3}{)}_{j}{\Theta }^{*}{{z}_{1}}_{j}+({b}_{4}{)}_{j}{\Theta }^{*}{{z}_{1}}_{j-1}+({b}_{5}{)}_{j}{\Theta }^{*}{{z}_{2}}_{j}+({b}_{6}{)}_{j}{\Theta }^{*}{{z}_{2}}_{j-1}\\ +({b}_{7}{)}_{j}{\Theta }^{*}{\theta }_{j}+({b}_{8}{)}_{j}{\Theta }^{*}{\theta }_{j-1}+({b}_{9}{)}_{j}{\Theta }^{*}({z}_{3}{)}_{j}+({b}_{10}{)}_{j}{\Theta }^{*}({z}_{3}{)}_{j-1}=({r}_{5}{)}_{j-\frac{1}{2}}.\end{array}$$

where

36 $$({r}_{1}{)}_{j-\frac{1}{2}}=-{f}_{j}+{f}_{j-1}+\frac{h}{2}({z}_{1}{)}_{j}+(({z}_{1}{)}_{j-1}),$$

37 $$({r}_{2}{)}_{j-\frac{1}{2}}=-({z}_{1}{)}_{j}+({z}_{1}{)}_{j-1}+\frac{h}{2}(({z}_{2}{)}_{j}+({z}_{2}{)}_{j-1}),$$

38 $$({r}_{3}{)}_{j-\frac{1}{2}}=-{\theta }_{j}+{\theta }_{j-1}+\frac{h}{2}(({z}_{3}{)}_{j}+({z}_{3}{)}_{j-1}),$$

39 $$({r}_{4}{)}_{j-\frac{1}{2}}=-h\left[{\alpha }^{*}\left(\frac{({z}_{2}{)}_{j}-({z}_{2}{)}_{j-1}}{h}\right)(1-{\beta }^{*}{\left(\frac{({z}_{2}{)}_{j}+({z}_{2}{)}_{j-1}}{2}\right)}^{2})\right]-h\left[\left[{\phi }_{b}(\left(\frac{{f}_{j}+{f}_{j-1}}{2}\right)\left(\frac{({z}_{2}{)}_{j}+({z}_{2}{)}_{j-1}}{2}\right)-{\left(\frac{({z}_{1}{)}_{j}+({z}_{1}{)}_{j-1}}{2}\right)}^{2})-K\frac{1}{{\phi }_{a}}\left(\frac{({z}_{1}{)}_{j}+({z}_{1}{)}_{j-1}}{2}\right)\right]\right],$$

40 $$({r}_{5}{)}_{j-\frac{1}{2}}=-h\left[\frac{\left(({z}_{3}{)}_{j}-({z}_{3}{)}_{j-1}\right)}{h}\left(1+\frac{1}{{\phi }_{d}}{P}_{r}{N}_{r}\right)\right]-h\frac{{\phi }_{c}}{{\phi }_{d}}{P}_{r}\left[\left(\frac{({f}_{j}+{f}_{j-1})(({z}_{3}{)}_{j}+({z}_{3}{)}_{j-1})}{4}\right)\right]+h\frac{{\phi }_{c}}{{\phi }_{d}}{P}_{r}\left[\left(\frac{({\theta }_{j}+{\theta }_{j-1})(({z}_{1}{)}_{j}+({z}_{1}{)}_{j-1})}{4}\right)\right]-h\frac{{\phi }_{c}}{{\phi }_{d}}{P}_{r}\left[\frac{{E}_{L}}{{\phi }_{a}{\phi }_{c}}{\left(\frac{({z}_{2}{)}_{j}+({z}_{2}{)}_{j-1}}{2}\right)}^{2}\right]=0.$$

The boundary condition becomes

41 $${\Theta }^{*}{f}_{0}=0,{\Theta }^{*}({z}_{1}{)}_{0}=0,{\Theta }^{*}({z}_{3}{)}_{0}=0,{\Theta }^{*}({z}_{1}{)}_{J}=0,{\Theta }^{*}{\theta }_{J}=0.$$

The boundary constraints should be fulfilled even in the event of whole iterations for the completion of the scheme discussed above. So, concern with our actual hypothesis, we apply the limit conditions discussed above for the maintenance of the correct values in each iteration.

### Stage 4: the block-tridiagonal array

A tridiagonal-block structure is used in linearized differential formulas (31)–(35). The method is written as follows in a matrix–vector

For $$j=1;$$

42 $${\Theta }^{*}{f}_{1}-{\Theta }^{*}{f}_{0}-\frac{1}{2}h({\Theta }^{*}({z}_{1}{)}_{1}+{\Theta }^{*}({z}_{1}{)}_{0})=({r}_{1}{)}_{1-\frac{1}{2}},$$

43 $${\Theta }^{*}({z}_{1}{)}_{1}-{\Theta }^{*}({z}_{1}{)}_{0}-\frac{1}{2}h({\Theta }^{*}({z}_{2}{)}_{1}+{\Theta }^{*}({z}_{2}{)}_{0})=({r}_{2}{)}_{1-\frac{1}{2}},$$

44 $${\Theta }^{*}{\theta }_{1}-{\Theta }^{*}{\theta }_{0}-\frac{1}{2}h({\Theta }^{*}({z}_{3}{)}_{1}+{\Theta }^{*}({z}_{3}{)}_{0})=({r}_{3}{)}_{1-\frac{1}{2}},$$

45 $$\begin{array}{l}({a}_{1}{)}_{1}{\Theta }^{*}{f}_{1}+({a}_{2}{)}_{1}{\Theta }^{*}{f}_{0}+({a}_{3}{)}_{1}{\Theta }^{*}{{z}_{{1}_{1}}}+({a}_{4}{)}_{1}{\Theta }^{*}{{z}_{{1}_{0}}}+({a}_{5}{)}_{1}{\Theta }^{*}{{z}_{{2}_{1}}}+({a}_{6}{)}_{1}{\Theta }^{*}{{z}_{{2}_{0}}}\\ +({a}_{7}{)}_{1}{\Theta }^{*}{\theta }_{j}+({a}_{8}{)}_{1}{\Theta }^{*}{\theta }_{0}+({a}_{9}{)}_{1}{\Theta }^{*}({z}_{3}{)}_{1}+({a}_{10}{)}_{1}{\Theta }^{*}({z}_{3}{)}_{0}=({r}_{4}{)}_{1-\frac{1}{2}},\end{array}$$

46 $$\begin{array}{l}({b}_{1}{)}_{1}{\Theta }^{*}{f}_{1}+({b}_{2}{)}_{1}{\Theta }^{*}{f}_{0}+({b}_{3}{)}_{1}{\Theta }^{*}{{z}_{{1}_{1}}}+({b}_{4}{)}_{1}{\Theta }^{*}{{z}_{{1}_{0}}}+({b}_{5}{)}_{1}{\Theta }^{*}{{z}_{{2}_{1}}}+({b}_{6}{)}_{1}{\Theta }^{*}{{z}_{{2}_{0}}}\\ +({b}_{7}{)}_{1}{\Theta }^{*}{\theta }_{1}+({b}_{8}{)}_{1}{\Theta }^{*}{\theta }_{0}+({b}_{9}{)}_{1}{\Theta }^{*}({z}_{3}{)}_{1}+({b}_{10}{)}_{1}{\Theta }^{*}({z}_{3}{)}_{0}=({r}_{5}{)}_{1-\frac{1}{2}}.\end{array}$$

In array arrangement,

47 $$\left[\begin{array}{lllll}0&\quad 0&\quad 1&\quad 0&\quad 0\\ -h/2&\quad 0&\quad 0&\quad -h/2&\quad 0\\ 0&\quad -h/2&\quad 0&\quad 0&\quad -h/2\\ ({a}_{2}{)}_{1}&\quad ({a}_{10}{)}_{1}&\quad ({a}_{3}{)}_{1}&\quad ({a}_{1}{)}_{1}&\quad ({a}_{9}{)}_{1}\\ ({b}_{2}{)}_{1}&\quad ({b}_{10}{)}_{1}& \quad({b}_{3}{)}_{1}&\quad ({b}_{1}{)}_{1}&\quad ({b}_{9}{)}_{1}\end{array}\right]\left[\begin{array}{l}{\Theta }^{*}({z}_{2}{)}_{0}\\ {\Theta }^{*}(\theta {)}_{0}\\ {\Theta }^{*}(f{)}_{1}\\ {\Theta }^{*}({z}_{2}{)}_{1}\\ {\Theta }^{*}({z}_{3}{)}_{1}\end{array}\right]+\left[\begin{array}{lllll}-h/2&\quad 0&\quad 0&\quad 0&\quad 0\\ 1&\quad 0&\quad 0&\quad 0&\quad 0\\ 0&\quad 1&\quad 0&\quad 0&\quad 0\\ ({a}_{5}{)}_{1}&\quad ({a}_{7}{)}_{1}&\quad 0&\quad 0&\quad 0\\ ({b}_{5}{)}_{1}&\quad ({b}_{7}{)}_{1}&\quad 0&\quad 0&\quad 0\end{array}\right]\left[\begin{array}{l}{\Theta }^{*}({z}_{1}{)}_{1}\\ {\Theta }^{*}(\theta {)}_{1}\\ {\Theta }^{*}(f{)}_{2}\\ {\Theta }^{*}({z}_{2}{)}_{2}\\ {\Theta }^{*}({z}_{3}{)}_{2}\end{array}\right]=\left[\begin{array}{l}({r}_{1}{)}_\frac{1}{2}\\ ({r}_{2}{)}_\frac{1}{2}\\ ({r}_{3}{)}_\frac{1}{2}\\ ({r}_{4}{)}_\frac{1}{2}\\ ({r}_{5}{)}_\frac{1}{2}\end{array}\right].$$

That is

48 $$[{A}_{1}][{\Theta }_{1}^{*}]+[{C}_{1}][{\Theta }_{2}^{*}]=[{r}_{1}].$$

For $$j=2;$$

49 $${\Theta }^{*}{f}_{2}-{\Theta }^{*}{f}_{1}-\frac{1}{2}h({\Theta }^{*}({z}_{1}{)}_{2}+{\Theta }^{*}({z}_{1}{)}_{1})=({r}_{1}{)}_{1-\frac{1}{2}},$$

50 $${\Theta }^{*}({z}_{1}{)}_{2}-{\Theta }^{*}({z}_{1}{)}_{1}-\frac{1}{2}h({\Theta }^{*}({z}_{2}{)}_{2}+{\Theta }^{*}({z}_{2}{)}_{1})=({r}_{2}{)}_{1-\frac{1}{2}},$$

51 $${\Theta }^{*}{\theta }_{1}-{\Theta }^{*}{\theta }_{0}-\frac{1}{2}h({\Theta }^{*}({z}_{3}{)}_{2}+{\Theta }^{*}({z}_{3}{)}_{1})=({r}_{3}{)}_{1-\frac{1}{2}},,$$

52 $$\begin{array}{l}({a}_{1}{)}_{2}{\Theta }^{*}{f}_{2}+({a}_{2}{)}_{2}{\Theta }^{*}{f}_{1}+({a}_{3}{)}_{2}{\Theta }^{*}{{z}_{{1}_{2}}}+({a}_{4}{)}_{2}{\Theta }^{*}{{z}_{{1}_{1}}}+({a}_{5}{)}_{2}{\Theta }^{*}{{z}_{{2}_{2}}}+({a}_{6}{)}_{2}{\Theta }^{*}{{z}_{{2}_{1}}}\\ +({a}_{7}{)}_{2}{\Theta }^{*}{\theta }_{2}+({a}_{8}{)}_{2}{\Theta }^{*}{\theta }_{1}+({a}_{9}{)}_{2}{\Theta }^{*}({z}_{3}{)}_{2}+({a}_{10}{)}_{2}{\Theta }^{*}({z}_{3}{)}_{1}=({r}_{4}{)}_{2-\frac{1}{2}},\end{array}$$

53 $$\begin{array}{l}({b}_{1}{)}_{2}{\Theta }^{*}{f}_{2}+({b}_{2}{)}_{2}{\Theta }^{*}{f}_{1}+({b}_{3}{)}_{2}{\Theta }^{*}{{z}_{{1}_{2}}}+({b}_{4}{)}_{2}{\Theta }^{*}{{z}_{{1}_{1}}}+({b}_{5}{)}_{2}{\Theta }^{*}{{z}_{{2}_{2}}}+({b}_{6}{)}_{2}{\Theta }^{*}{{z}_{{2}_{1}}}\\ +({b}_{7}{)}_{2}{\Theta }^{*}{\theta }_{2}+({b}_{8}{)}_{2}{\Theta }^{*}{\theta }_{1}+({b}_{9}{)}_{2}{\Theta }^{*}({z}_{3}{)}_{2}+({b}_{10}{)}_{2}{\Theta }^{*}({z}_{3}{)}_{1}=({r}_{5}{)}_{2-\frac{1}{2}}.\end{array}$$

In array arrangement,

54 $$\left[\begin{array}{lllll}0& 0& -1& 0& 0\\ 0& 0& 0& -h/2& 0\\ 0& 0& 0& 0& -h/2\\ 0& 0& ({a}_{4}{)}_{2}& ({a}_{2}{)}_{2}& ({a}_{10}{)}_{2}\\ 0& 0& ({b}_{4}{)}_{2}& ({b}_{2}{)}_{2}& ({b}_{10}{)}_{2}\end{array}\right]\left[\begin{array}{l}{\Theta }^{*}({z}_{2}{)}_{0}\\ {\Theta }^{*}(\theta {)}_{0}\\ {\Theta }^{*}(f{)}_{1}\\ {\Theta }^{*}({z}_{2}{)}_{1}\\ {\Theta }^{*}({z}_{3}{)}_{1}\end{array}\right]+\left[\begin{array}{lllll}-h/2& 0& 1& 0& 0\\ -1& 0& 0& -h/2& 0\\ 0& -1& 0& 0& -h/2\\ ({a}_{6}{)}_{2}& ({a}_{8}{)}_{2}& ({a}_{3}{)}_{2}& ({a}_{1}{)}_{2}& ({a}_{9}{)}_{2}\\ ({b}_{6}{)}_{2}& ({b}_{8}{)}_{2}& ({b}_{3}{)}_{2}& ({b}_{1}{)}_{2}& ({b}_{9}{)}_{2}\end{array}\right]\left[\begin{array}{l}{\Theta }^{*}({z}_{1}{)}_{1}\\ {\Theta }^{*}(\theta {)}_{1}\\ {\Theta }^{*}(f{)}_{2}\\ {\Theta }^{*}({z}_{2}{)}_{2}\\ {\Theta }^{*}({z}_{3}{)}_{2}\end{array}\right]+\left[\begin{array}{lllll}-h/2& 0& 1& 0& 0\\ 1& 0& 0& -h/2& 0\\ 0& 1& 0& 0& -h/2\\ ({a}_{5}{)}_{2}& ({a}_{7}{)}_{2}& 0& 0& 0\\ ({b}_{5}{)}_{2}& ({b}_{7}{)}_{2}& 0& 0& 0\end{array}\right]\left[\begin{array}{l}{\Theta }^{*}({z}_{1}{)}_{1}\\ {\Theta }^{*}(\theta {)}_{1}\\ {\Theta }^{*}(f{)}_{2}\\ {\Theta }^{*}({z}_{2}{)}_{2}\\ {\Theta }^{*}({z}_{3}{)}_{2}\end{array}\right]=\left[\begin{array}{l}({r}_{1}{)}_\frac{3}{2}\\ ({r}_{2}{)}_\frac{3}{2}\\ ({r}_{3}{)}_\frac{3}{2}\\ ({r}_{4}{)}_\frac{3}{2}\\ ({r}_{5}{)}_\frac{3}{2}\end{array}\right].$$

That is

55 $$[{B}_{2}][{\Theta }_{1}^{*}]+[{A}_{2}][{\Theta }_{2}^{*}]+[{C}_{2}][{\Theta }_{3}^{*}]=[{r}_{2}].$$

For $$j=J-1;$$

56 $${\Theta }^{*}{f}_{J-1}-{\Theta }^{*}{f}_{J-2}-\frac{1}{2}h({\Theta }^{*}({z}_{1}{)}_{J-1}+{\Theta }^{*}{{z}_{1}}_{J-2})=({r}_{1}{)}_{J-1-\frac{1}{2}},$$

57 $${\Theta }^{*}({z}_{1}{)}_{J-1}-{\Theta }^{*}({z}_{1}{)}_{J-2}-\frac{1}{2}h({\Theta }^{*}({z}_{2}{)}_{J-1}+{\Theta }^{*}({z}_{2}{)}_{J-2})=({r}_{2}{)}_{J-1-\frac{1}{2}},$$

58 $${\Theta }^{*}{\theta }_{J-1}-{\Theta }^{*}{\theta }_{J-2}-\frac{1}{2}h({\Theta }^{*}({z}_{3}{)}_{J-1}+{\Theta }^{*}({z}_{3}{)}_{J-2})=({r}_{3}{)}_{J-1-\frac{1}{2}},$$

59 $$\begin{array}{l}({a}_{1}{)}_{J-1}{\Theta }^{*}{f}_{J-1}+({a}_{2}{)}_{J-1}{\Theta }^{*}{f}_{J-2}+({a}_{3}{)}_{J-1}{\Theta }^{*}{{z}_{1}}_{J-1}+({a}_{4}{)}_{J-1}{\Theta }^{*}{{z}_{1}}_{J-2}\\ +({a}_{5}{)}_{J-1}{\Theta }^{*}{{z}_{2}}_{j}+({a}_{6}{)}_{J-1}{\Theta }^{*}{{z}_{2}}_{J-2}+({a}_{7}{)}_{J-1}{\Theta }^{*}{\theta }_{J-1}+({a}_{8}{)}_{J-1}{\Theta }^{*}{\theta }_{J-2}\\ +({a}_{9}{)}_{J-1}{\Theta }^{*}({z}_{3}{)}_{J-1}+({a}_{10}{)}_{J-1}{\Theta }^{*}({z}_{3}{)}_{J-2}=({r}_{4}{)}_{J-1-\frac{1}{2}},\end{array}$$

60 $$\begin{array}{l}({b}_{1}{)}_{J-1}{\Theta }^{*}{f}_{J-1}+({b}_{2}{)}_{J-1}{\Theta }^{*}{f}_{J-2}+({b}_{3}{)}_{J-1}{\Theta }^{*}{{z}_{1}}_{J-1}+({b}_{4}{)}_{J-1}{\Theta }^{*}{{z}_{1}}_{J-2}\\ +({b}_{5}{)}_{J-1}{\Theta }^{*}{{z}_{2}}_{J-1}+({b}_{6}{)}_{J-1}{\Theta }^{*}{{z}_{2}}_{J-2}+({b}_{7}{)}_{J-1}{\Theta }^{*}{\theta }_{J-1}+({b}_{8}{)}_{J-1}{\Theta }^{*}{\theta }_{J-2}\\ +({b}_{9}{)}_{J-1}{\Theta }^{*}({z}_{3}{)}_{J-1}+({b}_{10}{)}_{J-1}{\Theta }^{*}({z}_{3}{)}_{J-2}=({r}_{5}{)}_{J-1-\frac{1}{2}}.\end{array}$$

In array arrangement,

61 $$\left[\begin{array}{lllll}0& 0& -1& 0& 0\\ 0& 0& 0& -h/2& 0\\ 0& 0& 0& 0& -h/2\\ 0& 0& ({a}_{4}{)}_{J-2}& ({a}_{2}{)}_{J-2}& ({a}_{10}{)}_{J/2}\\ 0& 0& ({b}_{4}{)}_{J-2}& ({b}_{2}{)}_{J-2}& ({b}_{10}{)}_{J-2}\end{array}\right]\left[\begin{array}{l}{\Theta }^{*}({z}_{2}{)}_{J-3}\\ {\Theta }^{*}(\theta {)}_{J-3}\\ {\Theta }^{*}(f{)}_{J-2}\\ {\Theta }^{*}({z}_{2}{)}_{J-2}\\ {\Theta }^{*}({z}_{3}{)}_{J-2}\end{array}\right]+\left[\begin{array}{lllll}-h/2& 0& 1& 0& 0\\ -1& 0& 0& -h/2& 0\\ 0& -1& 0& 0& -h/2\\ ({a}_{6}{)}_{J-2}& ({a}_{8}{)}_{J-2}& ({a}_{3}{)}_{J-2}& ({a}_{1}{)}_{J-2}& ({a}_{9}{)}_{J-2}\\ ({b}_{6}{)}_{J-2}& ({b}_{8}{)}_{J-2}& ({b}_{3}{)}_{J-2}& ({b}_{1}{)}_{J-2}& ({b}_{9}{)}_{J-2}\end{array}\right]\left[\left[\begin{array}{l}{\Theta }^{*}({z}_{2}{)}_{J-2}\\ {\Theta }^{*}(\theta {)}_{J-2}\\ {\Theta }^{*}(f{)}_{J-1}\\ {\Theta }^{*}({z}_{2}{)}_{J-1}\\ {\Theta }^{*}({z}_{3}{)}_{J-1}\end{array}\right]\right]+\left[\begin{array}{lllll}-h/2& 0& 0& 0& 0\\ 1& 0& 0& 0& 0\\ 0& 1& 0& 0& 0\\ ({a}_{5}{)}_{J-2}& ({a}_{9}{)}_{J-2}& 0& 0& 0\\ ({b}_{5}{)}_{J-2}& ({b}_{9}{)}_{J-2}& 0& 0& 0\end{array}\right]\left[\begin{array}{l}{\Theta }^{*}({z}_{1}{)}_{J-1}\\ {\Theta }^{*}(\theta {)}_{J-1}\\ {\Theta }^{*}(f{)}_{J}\\ {\Theta }^{*}({z}_{2}{)}_{J}\\ {\Theta }^{*}({z}_{3}{)}_{J}\end{array}\right]=\left[\begin{array}{l}({r}_{1}{)}_{(J-1)-\frac{1}{2}}\\ ({r}_{2}{)}_{(J-1)-\frac{1}{2}}\\ ({r}_{3}{)}_{(J-1)-\frac{1}{2}}\\ ({r}_{4}{)}_{(J-1)-\frac{1}{2}}\\ ({r}_{5}{)}_{(J-1)-\frac{1}{2}}\end{array}\right].$$

That is

62 $$[{B}_{J-1}][{\Theta }_{J-2}^{*}]+[{A}_{J-1}][{\Theta }_{J-1}^{*}]+[{C}_{J-1}][{\Theta }_{J}^{*}]=[{r}_{J-1}].$$

For $$j=J;$$

63 $${\Theta }^{*}{f}_{J}-{\Theta }^{*}{f}_{J-1}-\frac{1}{2}h({\Theta }^{*}({z}_{1}{)}_{J}+{\Theta }^{*}({z}_{1}{)}_{J-1})=({r}_{1}{)}_{J-\frac{1}{2}},$$

64 $${\Theta }^{*}({z}_{1}{)}_{J}-{\Theta }^{*}({z}_{1}{)}_{J-1}-\frac{1}{2}h({\Theta }^{*}({z}_{2}{)}_{J}+{\Theta }^{*}({z}_{2}{)}_{J-1})=({r}_{2}{)}_{J-\frac{1}{2}},$$

65 $${\Theta }^{*}{\theta }_{J}-{\Theta }^{*}{\theta }_{J-1}-\frac{1}{2}h({\Theta }^{*}({z}_{3}{)}_{J}+{\Theta }^{*}({z}_{3}{)}_{J-1})=({r}_{3}{)}_{J-\frac{1}{2}},$$

66 $$\begin{array}{l}({a}_{1}{)}_{J}{\Theta }^{*}{f}_{J}+({a}_{2}{)}_{J}{\Theta }^{*}{f}_{J-1}+({a}_{3}{)}_{J}{\Theta }^{*}{{z}_{1}}_{J}+({a}_{4}{)}_{J}{\Theta }^{*}{{z}_{1}}_{J-1}+({a}_{5}{)}_{J}{\Theta }^{*}{{z}_{2}}_{J}+({a}_{6}{)}_{J}{\Theta }^{*}{{z}_{2}}_{J-1}\\ +({a}_{7}{)}_{J}{\Theta }^{*}{\theta }_{J}+({a}_{8}{)}_{J}{\Theta }^{*}{\theta }_{J-1}+({a}_{9}{)}_{J}{\Theta }^{*}({z}_{3}{)}_{J}+({a}_{10}{)}_{J}{\Theta }^{*}({z}_{3}{)}_{J-1}=({r}_{4}{)}_{J-\frac{1}{2}},\end{array}$$

67 $$\begin{array}{l}({b}_{1}{)}_{J}{\Theta }^{*}{f}_{J}+({b}_{2}{)}_{J}{\Theta }^{*}{f}_{J-1}+({b}_{3}{)}_{J}{\Theta }^{*}{{z}_{1}}_{J}+({b}_{4}{)}_{J}{\Theta }^{*}{{z}_{1}}_{J-1}+({b}_{5}{)}_{J}{\Theta }^{*}{{z}_{2}}_{J}+({b}_{6}{)}_{J}{\Theta }^{*}{{z}_{2}}_{J-1}\\ +({b}_{7}{)}_{J}{\Theta }^{*}{\theta }_{J}+({b}_{8}{)}_{J}{\Theta }^{*}{\theta }_{J-1}+({b}_{9}{)}_{J}{\Theta }^{*}({z}_{3}{)}_{J}+({b}_{10}{)}_{J}{\Theta }^{*}({z}_{3}{)}_{J-1}=({r}_{5}{)}_{J-\frac{1}{2}}.\end{array}$$

In matrix form,

68 $$\left[\begin{array}{lllll}-h/2& 0& 1& 0& 0\\ -1& 0& 0& -h/2& 0\\ 0& -1& 0& 0& -h/2\\ ({a}_{6}{)}_{1}& ({a}_{8}{)}_{1}& ({a}_{3}{)}_{1}& ({a}_{1}{)}_{1}& ({a}_{9}{)}_{1}\\ ({b}_{6}{)}_{1}& ({b}_{8}{)}_{1}& ({b}_{3}{)}_{1}& ({b}_{1}{)}_{1}& ({b}_{9}{)}_{1}\end{array}\right]\left[\begin{array}{l}{\Theta }^{*}({z}_{2}{)}_{0}\\ {\Theta }^{*}(\theta {)}_{0}\\ {\Theta }^{*}(f{)}_{1}\\ {\Theta }^{*}({z}_{2}{)}_{1}\\ {\Theta }^{*}({z}_{3}{)}_{1}\end{array}\right]+\left[\begin{array}{lllll}-h/2& 0& 1& 0& 0\\ -1& 0& 0& -h/2& 0\\ 0& -1& 0& 0& -h/2\\ ({a}_{6}{)}_{J-2}& ({a}_{8}{)}_{J-2}& ({a}_{3}{)}_{J-2}& ({a}_{1}{)}_{J-2}& ({a}_{9}{)}_{J-2}\\ ({b}_{6}{)}_{J-2}& ({b}_{8}{)}_{J-2}& ({b}_{3}{)}_{J-2}& ({b}_{1}{)}_{J-2}& ({b}_{9}{)}_{J-2}\end{array}\right]\left[\begin{array}{l}{\Theta }^{*}({z}_{2}{)}_{J-2}\\ {\Theta }^{*}(\theta {)}_{J-2}\\ {\Theta }^{*}(f{)}_{J-1}\\ {\Theta }^{*}({z}_{2}{)}_{J-1}\\ {\Theta }^{*}({z}_{3}{)}_{J-1}\end{array}\right]=\left[\begin{array}{l}({r}_{1}{)}_\frac{1}{2}\\ ({r}_{2}{)}_\frac{1}{2}\\ ({r}_{3}{)}_\frac{1}{2}\\ ({r}_{4}{)}_\frac{1}{2}\\ ({r}_{5}{)}_\frac{1}{2}\end{array}\right].$$

That is

69 $$[{B}_{J}][{\Theta }_{J-1}^{*}]+[{A}_{J}][{\Theta }_{J}^{*}]=[{r}_{J}].$$

### Stage 5: the bulk eliminated technique

By using Eqs. (42)–(67), the tridiagonal-block array is gained as follows,

70 $$R\Pi =p,$$

where

71 $$R=\left[\begin{array}{llllll}{A}_{1}& {C}_{1}& & & & \\ {B}_{2}& {A}_{2}& {C}_{2}& & & \\ & \ddots & \ddots & \ddots & & \\ & & \ddots & \ddots & \ddots & \\ & & & {B}_{J-1}& {A}_{J-1}& {C}_{J-1}\\ & & & & {B}_{J}& {A}_{J}\end{array}\right],{\Theta }^{*}=\left[\begin{array}{l}{\Theta }_{1}^{*}\\ {\Theta }_{2}^{*}\\ \vdots \\ {\Theta }_{j-1}^{*}\\ {\Theta }_{j}^{*}\end{array}\right],p=\left[\begin{array}{l}({r}_{1}{)}_{j-\frac{1}{2}}\\ ({r}_{2}{)}_{j-\frac{1}{2}}\\ \vdots \\ ({r}_{J-1}{)}_{j-\frac{1}{2}}\\ ({r}_{J}{)}_{j-\frac{1}{2}}\end{array}\right].$$

Here $R$ increases the $J\times J$ tridiagonal block array for all heavy sizes of $5\times 5$, while, ${\Theta }^{*}$ and $p$ are $J\times 1$ order columns vector. The factorization method LU is applied to obtain the ${\Theta }^{*}$ solution. Array $R$ must be selected in a way that provides us a nonsingular matrix such that factoring can be done easily. Whereas $R{\Theta }^{*}=p$ works on the vector directly to the tridiagonal block array $R$, to generate another vector $p$. The tridiagonal $R$ factorization bulk is extra classified into the triangular matrices lower and upper, i.e.,$R=LU$ can be expressed as $LU{\Theta }^{*}=p$, so let $U{\Theta }^{*}=y$ generates $Ly=p$, which provides $y$ solution is once again connected into $U{\Theta }^{*}=y$ to answer for ${\Theta }^{*}$. Since we deal with triangular arrays, replacing is the path forward.

## Code verification

On the other, by measuring the heat transmission rate outcomes from the current technique against the recent results available in the literature^54–57^, the method's validity was evaluated. Table 4 summarises the comparing of reliabilities current during the researches. Nevertheless, the outcomes of the current examination are exceedingly accurate.

**Table 4:** Comparing of $- \theta^{\prime}\left( 0 \right)$ values with $P_{r}$, when $\phi = 0$, $\phi_{hnf} = 0$, ${\text{E}}_{L} = 0$, ${\Lambda }_{L} = 0$, $N_{L} = 0$, $S = 0$ and $G_{L} = 0$.

$P_{r}$	Ref.^54^	Ref.^55^	Ref.^56^	Ref.^57^	Present
72 × 10^–2^	08086 × 10^–4^	08086 × 10^–4^	080863135 × 10^–8^	080876122 × 10^–8^	080876181 × 10^–8^
1 × 10^0^	1 × 10^0^	1 × 10^0^	1 × 10^0^	1 × 10^0^	1 × 10^0^
3 × 10^0^	19,237 × 10^–4^	19,236 × 10^–4^	192,368,259 × 10^–8^	192,357,431 × 10^–8^	192,357,420 × 10^–8^
7 × 10^0^	30,723 × 10^–4^	30,722 × 10^–4^	307,225,021 × 10^–8^	307,314,679 × 10^–8^	307,314,651 × 10^–8^
10 × 10^0^	37,207 × 10^–4^	37,006 × 10^–4^	372,067,390 × 10^–8^	372,055,436 × 10^–8^	372,055,429 × 10^–8^

The strategy of finite differences was adopted by Ishak et al.^54, 55^ to explore the solution of the model considered. The entropy study by HAM (homotopy analysis method) for time-dependent magneto-nanofluid was proposed by Abolbashari et al.^56^ (HAM). Das et al.^57^ resolved with the RK Fehlberg technique the unsteadiness status of the controlling formulas. Compared to previous ones, KBM applied here provides very precise performance.

## Entropy analysis

Porous media generally increase the entropy of the system^58–61^ described the nanofluid entropy production by:

72 $${E}_{G}=\frac{{k}_{hnf}}{{{\yen }}_{\infty }^{2}}\left\{{\left(\frac{\partial {\yen }}{\partial y}\right)}^{2}+\frac{16}{3}\frac{{\sigma }^{*}{{\yen }}_{\infty }^{3}}{{\kappa }^{*}{\nu }_{f}(\rho {C}_{p}{)}_{f}}{\left(\frac{\partial {\yen }}{\partial y}\right)}^{2}\right\}+\frac{{\mu }_{hnf}}{{{\yen }}_{\infty }}{\left(\frac{\partial {v}_{1}}{\partial y}\right)}^{2}+\frac{{\mu }_{hnf}{{v}_{1}}^{2}}{k{{\yen }}_{\infty }}.$$

The non-dimensional formulation of entropy analysis is as follows^62–66^

73 $$N_{G} = \frac{{\yen_{\infty }^{2} b^{2} E_{G} }}{{k_{f} \left( {\yen_{w} - \yen_{\infty } } \right)^{2} }}.$$

By formula (9), the non-dimensionalentropy formula is:

74 $$N_{G} = R_{e} \left[ {\phi_{d} \left( {1 + N_{r} } \right)\theta ^{{\prime}{2}} + \frac{1}{{\phi_{a} }}\frac{{B_{r} }}{{\Omega }}\left( {f^{{\prime\prime}{2}} + Kf^{{\prime}{2}} } \right)} \right],$$

Here $R_{e} = \frac{{U_{w} b^{2} }}{{\nu_{f} x}}$ is the Reynolds number, $B_{r} = \frac{{\mu_{f} U_{w}^{2} }}{{k_{f} \left( {\yen_{w} - \yen_{\infty } } \right)}}$. signifies the Brinkman amount and ${\Omega } = \frac{{_{w} - \yen_{\infty } }}{{\yen_{\infty } }}$ symbols the dimensionless gradient of the temperature.

## Results and discussion

**Figure 5:**
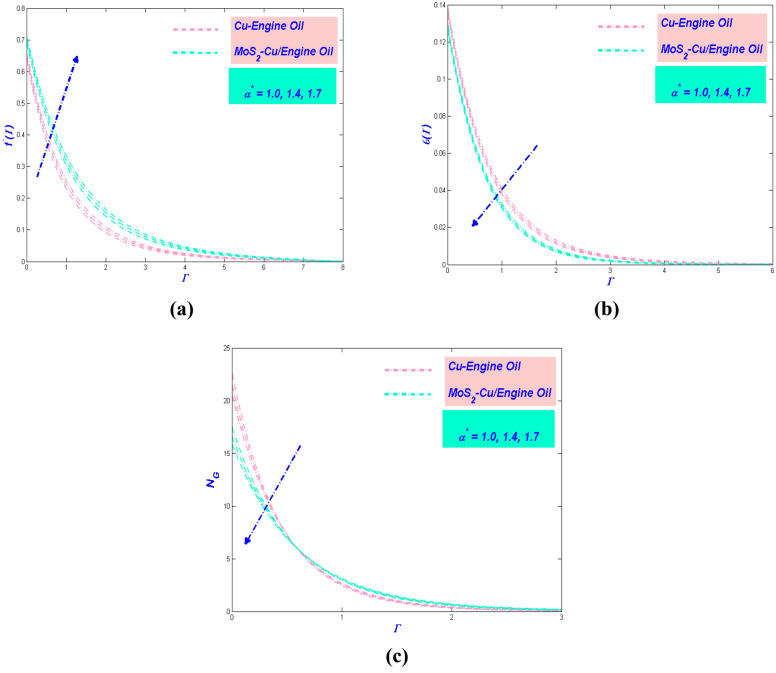
(**a**) Velocity, (**b**) temperature and (**c**) entropy variation versus $\alpha^{*}$.

**Figure 6:**
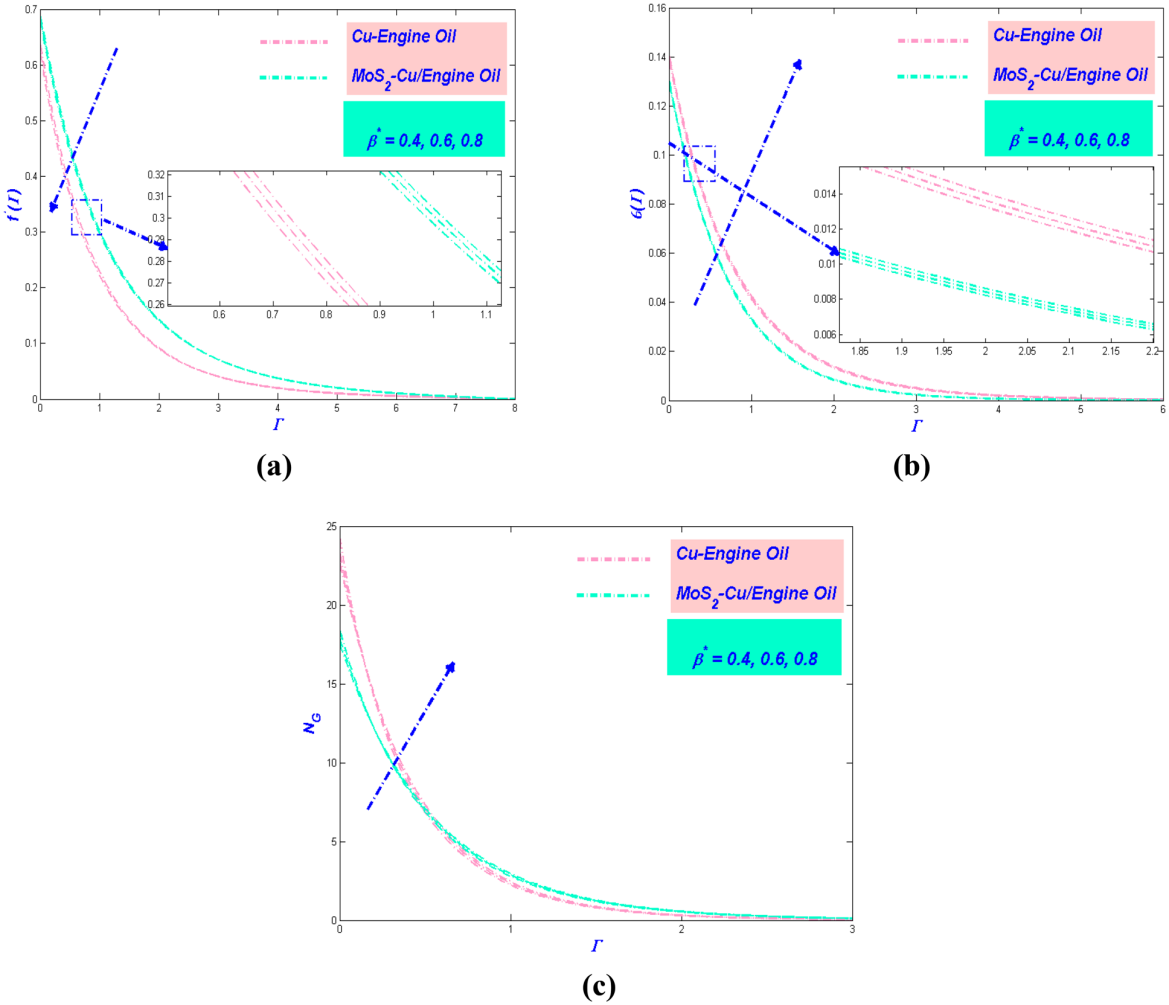
(**a**) Velocity, (**b**) temperature and (**c**) entropy variation versus $\beta^{*}$.

**Figure 7:**
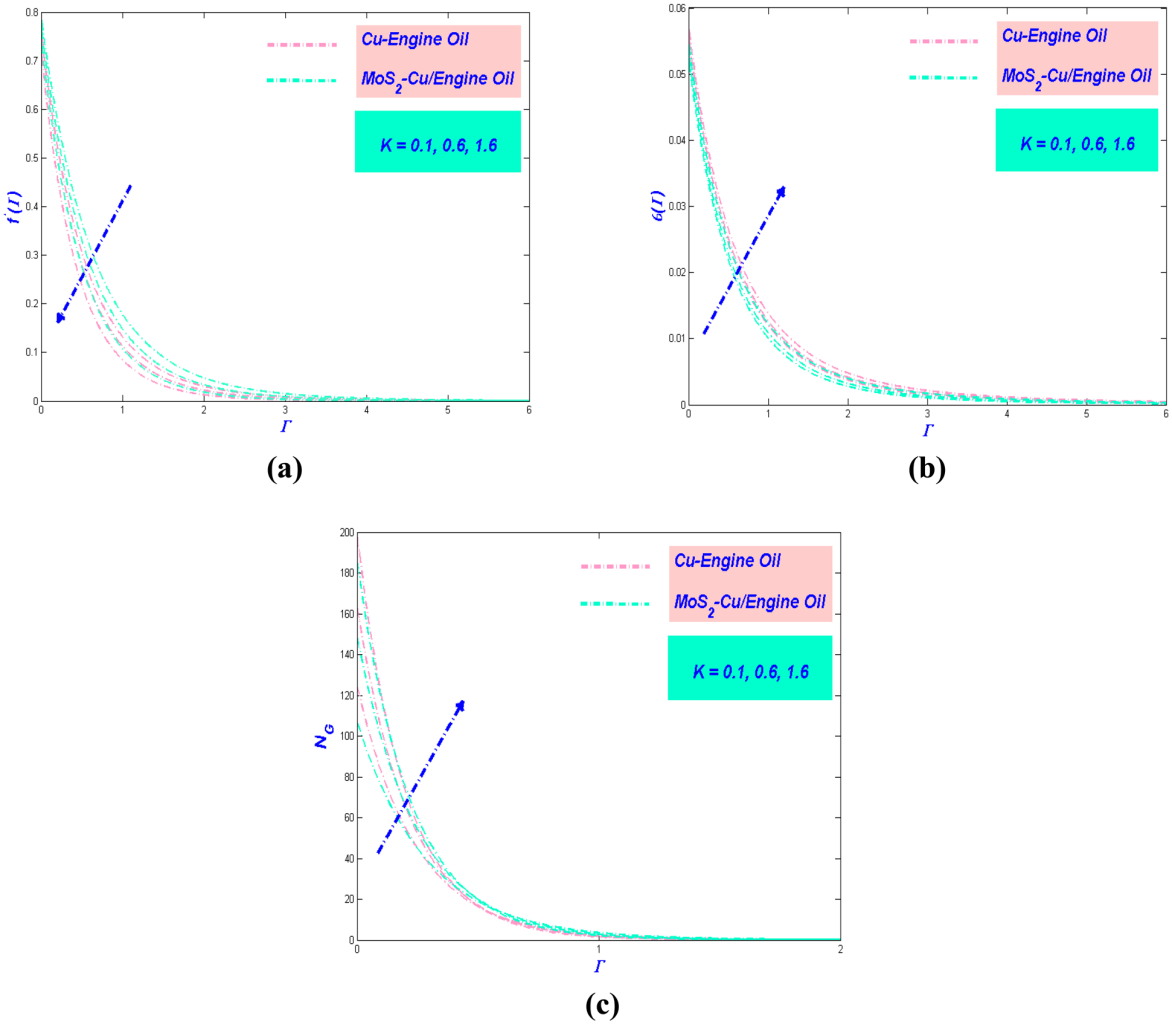
(**a**) Velocity, (**b**) temperature, and (**c**) entropy change with $K$.

**Figure 8:**
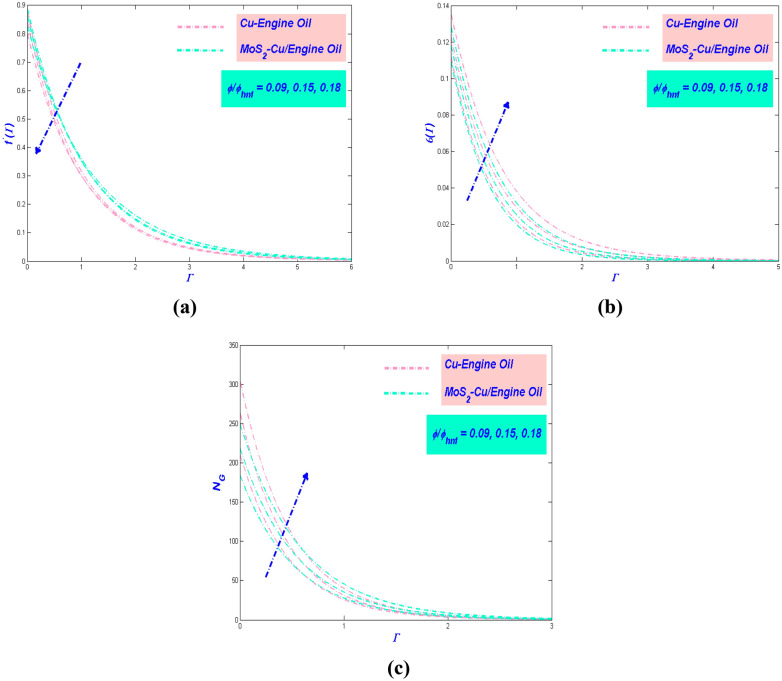
(**a**) Velocity, (**b**) temperature and (**c**) entropy versus $\phi$ and $\phi_{hnf}$.

**Figure 9:**
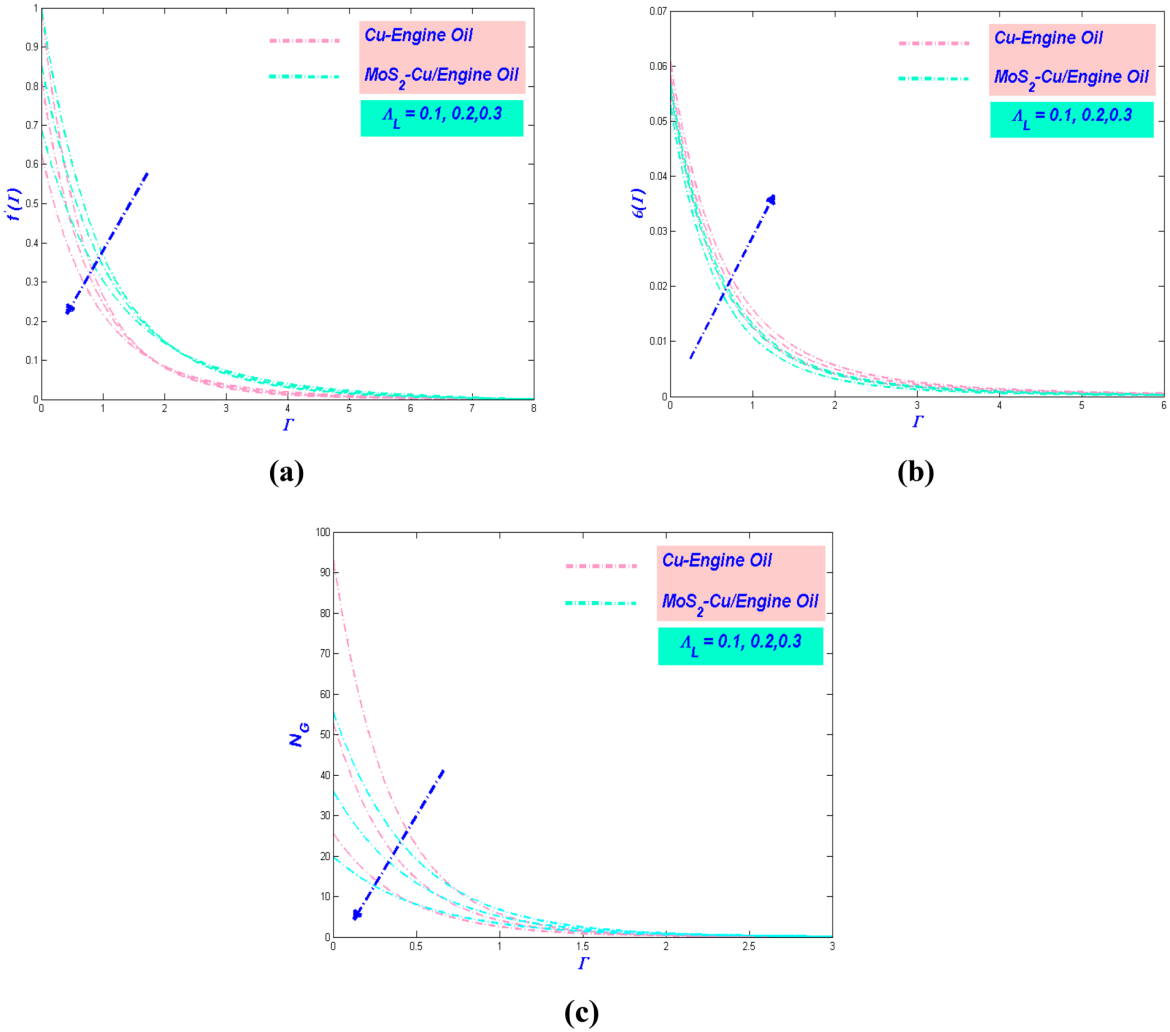
(**a**) Velocity, (**b**) temperature and (**c**) entropy variations with ${\Lambda }_{L}$.

**Figure 10:**
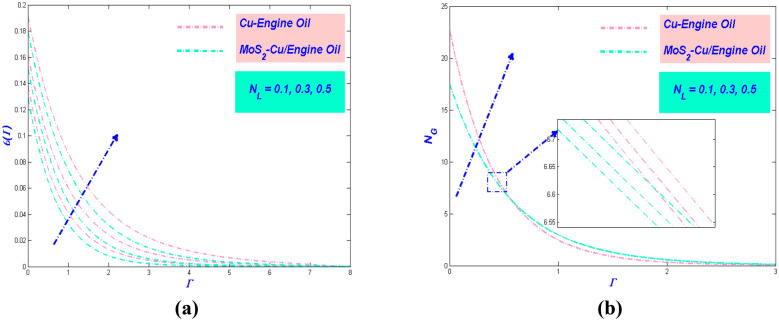
(**a**) Temperature and (**b**) entropy variations versus $N_{L}$.

**Figure 11:**
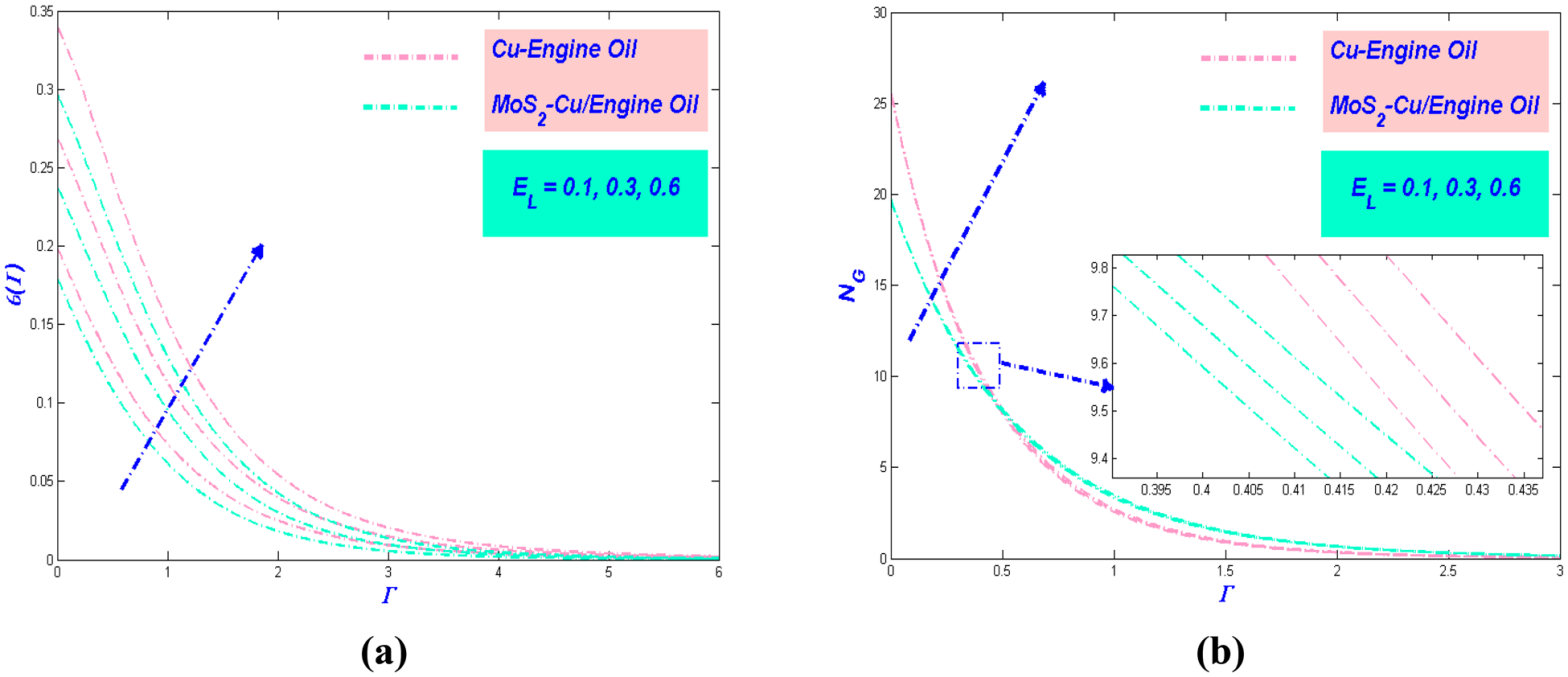
(**a**) Temperature and (**b**) entropy variations with $E_{L}$.

**Figure 12:**
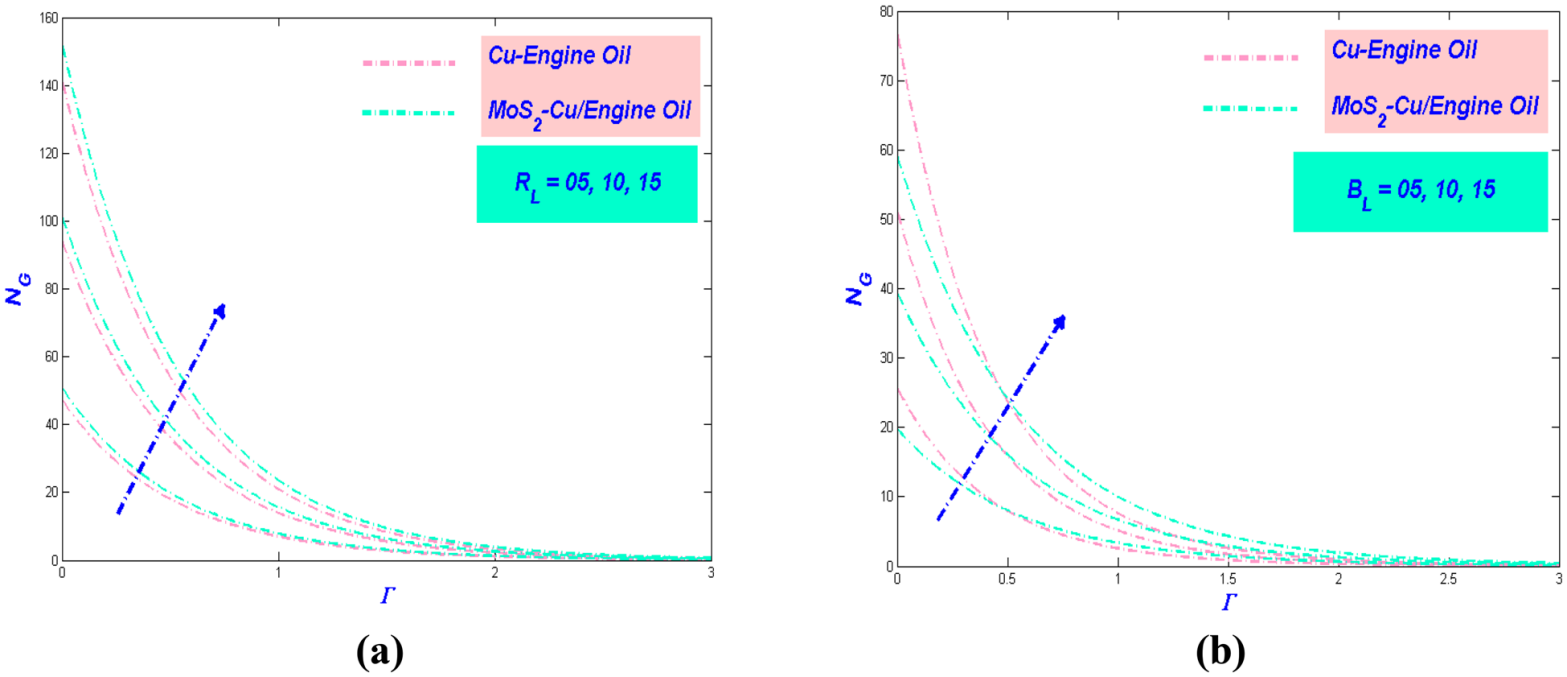
Entropy variations versus (**a**) $R_{e}$ and (**b**) $B_{r}$.

The following discussion is based onhe numerical results obtained from the model mentioned in the last part. The potential parameters $\alpha^{*}$, $\beta^{*} ,\;K$, $\phi$, ${\Lambda }_{L} S$, ${\text{E}}_{L} ,\;N_{L}$, $G_{L}$, $R_{L}$ and $B_{L}$ are assumed in this section. These variables reveal the physical aspects, including, swiftness, temperature, and entropy, of the dimensionless values in Figs. 5, 6, 7, 8, 9, 10, 11 and 12. For Cu–EO trivial P-ENF and MoS_2_–Cu/E0 non-Newtonian P-EHNF, the results are extracted. Table 5 demonstrates the drag force coefficients and temperature change. The extra values were $\alpha^{*} = 1.0,\;{ }\beta^{*} = 0.4$, $K = 0.1$, $\phi = 0.18$, $\phi_{Ms} = 0.09$, ${\Lambda }_{L} = 0.3$, $S = 0.4$, $N_{L} = 0.3$, ${\text{E}}_{L} = 0.3,\;G_{L} = 0.3$, $R_{L} = 5$ and $B_{L} = 5$. In order to have insight into the physical problem, these results are debated concisely in the following subsection.

**Table 5:** Values of $C_{f} Re_{x}^{1/2}$ and $Nu_{x} Re_{x}^{ - 1/2}$ for $P_{r} = 6450$.

$\alpha^{*}$	$\beta^{*}$	$K$	$\phi$	$\phi_{Ms}$	${\Lambda }_{L}$	$S$	$N_{L}$	$G_{L}$	$E_{L}$	$C_{f} Re_{x}^{\frac{1}{2}}$ Cu–EO	$C_{f} Re_{x}^{\frac{1}{2}}$ MoS_2_–Cu/ EO	$Nu_{x} Re_{x}^{{\frac{ - 1}{2}}}$ Cu–EO	$Nu_{x} Re_{x}^{{\frac{ - 1}{2}}}$ MoS_2_–Cu/EO	Relative %$\frac{{Nu_{{\left( {Ms - Cu/EO} \right) - Nu_{{\left( {Cu} \right)}} }} }}{{Nu_{{\left( {Ms - Cu/EO} \right)}} }}$
1.0	0.4	0.1	0.18	0.09	0.3	0.4	0.3	0.3	0.3	4.3045	5.1062	3.7097	3.8554	3.7%
1.4										4.3320	5.1316	3.7363	3.8903	3.9%
1.7										4.3517	5.1601	3.7523	3.9245	4.3%
	0.4									4.3045	5.1062	3.7097	3.8554	3.7%
	0.6									4.2825	5.0988	3.6712	3.8320	4.1%
	0.8									4.2571	5.0221	3.6542	3.8163	4.2%
		0.1								4.3045	5.1062	3.7097	3.8554	3.7%
		0.6								4.3390	5.1336	3.7215	3.8871	4.2%
		1.6								4.3551	5.1754	3.7563	3.9282	4.3%
			0.09							4.2436	–	3.6484	–	–
			0.15							4.2809	–	3.6796	–	3.6%
			0.18							4.3045	–	3.7097	–	3.7%
				0.0						–	4.2436	–	3.6484	–
				0.06						–	5.0926	–	3.8207	3.6%
				0.09						–	5.1062	–	3.8554	3.7%
					0.1					4.3534	5.1288	3.7616	3.8954	3.4%
					0.2					4.3382	5.1163	3.7325	3.8711	3.5%
					0.3					4.3045	5.1062	3.7097	3.8554	3.7%
						0.2				4.2715	5.0958	3.6752	3.8235	3.8%
						0.4				4.3045	5.1062	3.7097	3.8554	3.7%
						0.6				4.3260	5.1266	3.7235	3.8831	4.1%
							0.1			4.3045	5.1062	3.6831	3.8167	3.5%
							0.3			4.3045	5.1062	3.7097	3.8554	3.7%
							0.5			4.3045	5.1062	3.7243	3.8895	4.2%
								0.1		4.3045	5.1062	3.6720	3.8040	3.4%
								0.3		4.3045	5.1062	3.7097	3.8554	3.7%
								0.5		4.3045	5.1062	3.7234	3.8959	4.4%
									0.1	4.3045	5.1062	3.6811	3.8105	3.3%
									0.3	4.3045	5.1062	3.7097	3.8554	3.7%
									0.6	4.3045	5.1062	3.7402	3.8978	4.0%

### Influence of Prandtl–Eyring parameter ${{\alpha}}^{*}$

The impact of $\alpha^{*}$ towards the velocity of the hybrid nanofluid has been clearly shown in Fig. 5a. It is noted that amplify $\alpha^{*}$ will quicken further the velocity motion. The physical motivation behind this phenomenon is the $\alpha^{*}$ causes deterioration in viscosity of the fluid where the resistance will lessening and instead upsurge the velocity of the fluid. Furthermore, the MoS_2_–Cu/EO has a greater thickness of boundary layer flow as compared to Cu–EO. The physical intention is the nanofluid devising a higher density influence compared to the hybrid nanofluid. In contrast, the temperature profile of the flow exposed declining manner as $\alpha^{*}$ enlarge, which has been depicted in Fig. 5b. The triggered of this decreased manner due to the velocity intensifications, and more heat can be transmitted faster. It is also being noticed that MoS_2_–Cu/EO has a lower temperature compared to Cu–EO. It is because the hybrid nanofluid has a lower thermal conductivity in comparison with nanofluid. It is worth considering the entropy variation in the influence of $\alpha^{*}$. Figure 5c illustrates the entropy reduced as $\alpha^{*}$ augmented. The reason behind this phenomenon is the hybrid nanofluid motion diminution due to lower temperature, which will distress the entropy of the system reduction. Moreover, as the heat transfer rate increases in Table 5, the thermal efficiency of PTSC will improve in a solar water pump.

### Influence of Prandtl–Eyring parameter ${{\beta}}^{*}$

The actions of the fluid velocity by the effect of $\beta^{*}$ being exposed in Fig. 6a. The figure reveals the velocity subsidence as $\beta^{*}$ proliferate. It is well known that $\beta^{*}$ varies inversely with the momentum diffusivity and $\beta^{*}$ also brings resistance to the hybrid nanofluid particle. Hence, $\beta^{*}$ will diminish the velocity of the flow. It is noticeable that Cu–EO has deficient speed compared to MoS_2_–Cu/EO. The density of Cu–EO is thicker than MoS_2_–Cu/EO, which makes the flow arduous to move. Moreover, Fig. 6b portrayed the variation of temperature with the implementation of $\beta^{*}$. It is apparent from the figure that the temperature boost as $\beta^{*}$ surge. The circumstance happens attributable to the flow velocity cutback; hence there has been depreciation in transmitting the heat from the surface. Therefore, the entropy of the system will intensify, as adorned in Fig. 6c. $\beta^{*}$ magnified the hindrance in the system, as a result, the entropy of the system growth.

### Effect of porous media variable ${{K}}$

The aftermath of $K$ beneficial to the velocity of the flow exhibited in Fig. 7a. The velocity depreciated when $K$ upsurged. It is acknowledged that the porosity will make the fluid flow pathway being separated hence shrinkage the velocity of the flow. In other words, the porosity will become an obstruction to the flow. The reduction of velocity will always be allied to temperature demeanor. Figure 7b exemplifies the consequence of $K$ concerning the temperature of the flow. The temperature is seen to augment as $K$ raise. The trend, as mentioned earlier detected in Fig. 7c. The same explanation is established for this behavior. The entropy of the system will be inflation as $K$ increased (Fig. 7c). This action cannot be avoided since the temperature profile is pictured as dilated. It is perceptible that the entropy for Cu–EO is quite similar to MoS_2_–Cu/EO. This anomaly occurs because the size of molecule hybrid and non-hybrid nanofluid is equivalent to each other.

### Effect of nanomolecules size $\phi$ and $\phi_{{{{hnf}}}}$

The consequence of the nano molecules size regarding velocity, temperature, and entropy profile is worth to be discussed. The numerical results for velocity variation are pointed out in Fig. 8a. The velocity is waring as the nano molecule size aggrandize. This decency takes place due to the surface area of the nano particles will be enhanced, and afterward, the density of hybrid nanofluid will be increased. Hence, it will produce a decline in the flow velocity. A noticeable, MoS_2_–Cu/EO has the greatest speed value when the size of nano molecules is so small. Hence, this showed that the dispersion of nano molecules would be optimum when the size is small enough. As seen in Fig. 8b, the temperature of the flow rises as the nano molecule size upsurge. The decline in the nano molecule size will make the nano molecules diffuse in the far-field flow because of the temperature difference. It will cause an increment in the thickness of the thermal boundary layer. The minimum value of the size of nano molecules invented that it can be used to produce the lowest temperature profile. The variation of the entropy obliging the nano molecule size is delineated in Fig. 8c. It indicated that the rise of nano molecule size would escalate the entropy profile. The entropy of Cu–EO is higher than MoS_2_–Cu/EO due to the hybrid nanofluid has a large amount of thermal conductivity as compared to the nanofluid.

### Effect of velocity slip variable ${{{\Lambda}}}_{{{L}}}$

Figure 9a embossed the velocity slip notorious the velocity of the flow. It is exposed that the velocity slip makes the velocity declining. It is expected that the velocity slip can create more disturbance and deaccelerate the fluid motion. In the physical sense, when the velocity slip is increased, the far-field velocity flow shows a decreasing behaviour; so, the applied forces to pull the stretching surface are reduced and cannot transfer the energy to the fluid. However, it is observed that MoS_2_–Cu/EO has the maximum velocity as compared to Cu–EO because of their significance in thermophysical properties of hybrid nanofluid. The temperature profile will be affected by the changes made by the velocity profile. Figure 9b illustrated the difference in the demeanor of temperature when velocity slips acting on the system. The depreciation of velocity will have affected the viscosity of the boundary layer to be more viscous. Hence it will have magnified the temperature of the flow. It is expected to see that the temperature of MoS_2_–Cu/EO is the lowest than Cu–EO since the hybrid nanofluid has less viscosity than the conventional nanofluid. However, a different case happens to the entropy variation for the effect of velocity slip. As seen in Fig. 9c, the entropy of the system abatement as the increment of velocity slips. These results are also being agreed by Turkylimazoglu^67^.

### Thermal radiative variable ${{N}}_{{{r}}}$ influence

Thermal radiation is significant because the basic factor that affects the temperature of the earth as a whole is balancing the heat difference between the coming solar radiations and the earth's outgoing thermal radiation. The increment of the thermal radiative variable will enhance the temperature variation, as depicted in Fig. 10a. In the physical sense, this increment can be justified by assuming the thermal radiation converse into electromagnetic energy, and as a result, the distance of radiate from the surface increases, which will eventually raise the temperature of the boundary layer flow. Hence, the thermal radiative variable plays a crucial role to check the temperature profile of the system. It is also beneficial to observe the performance of entropy with the repercussion of the thermal radiative variable. Figure 10b embellished the augmentation of the entropy in the interest of strengthening the thermal radiative variable. The reasoning for this happening by the goodness of the existence of the irreparable nature of the heat transfer that takes place in the system. Also, the increasing behaviour of the Nussult number Table 5 will cause to increase in the thermal efficacy and performance of PTSC in SWP.

### Effect of Eckert number ${{E}}_{{{L}}}$

The Eckert number $E_{L}$ constantly referring to the kinetic energy of the fluid enthalpy. Based on Fig. 11a, the increment of $E_{L}$ influenced the temperature profile to upsurge. This behavior arises cause of the action of dissipation which will involve the internal friction of the flow. Further, it will cause self-heating and enhance the temperature of the system. The temperature variation for MoS_2_–Cu/EO has a lower value than Cu–EO due to its physical properties. Meanwhile, the entropy of the system showed increment when $E_{L}$ boost up, as shown in Fig. 11b. Entropy production effect of increasing $E_{L}$ is evident at the stretched sheet surface. However, in the central flow zone, it does not have a significant influence. Still, the hybrid nanofluid has the lowest entropy in the system. Furthermore, as the heat transfer rate increases in Table 5, the thermal efficiency of PTSC will improve in SWP.

### *R*_*e*_ and *B*_*r*_ influences on entropy rate

It is meaningfulness to point the efficacy of the Reynolds number propitious the fluid flow. It is widely known that can directly affect entropy production. This evidence can be shown in Fig. 12a, which showed the amplify of makes the entropy generation augmentation further. This twist is because of augmentation in fluid friction and the thermal boundary layer thickness. This will also create a disturbance in the fluid flow and changes the flow into disorganization. Figure 12b details the entropy profile fluctuation against the Brinkman number's multiple values. The number of Brinkman is the difference between heat energy produced by the transmission of molecules with the feature of viscous dislocation and heat. Theoretically, the dominant aspect in the heat produced by viscous dislocation causes a decline for the growing values of Brinkman numbers, which leads to an augmentation in the rate of entropy generation.

In the engineering interest, the numerical values of some physical quantities of different values of the pertinent parameters are shown in Table 5. It is perceived that the skin friction coefficient $C_{f} Re_{x}^{1/2}$ aggrandize as the increment of $\alpha^{*} , K,\phi ,\phi_{Ms}$ and $S$ parameters. Unfortunately, the enhanced $\beta^{*}$ and ${\Lambda }_{L}$ parameters affected $C_{f} Re_{x}^{1/2}$ to depreciate. On the other hand, the relative local Nusselt number $Nu_{x} Re_{x}^{ - 1/2}$ (in percentage) for hybrid nanofluid demonstrated an intensify with the rise of $\alpha^{*} , \;\beta^{*} ,\; K$ and ${\Lambda }_{L}$ parameters. While the $\phi , \;\phi_{Ms} , \;S, \;N_{L} , \;G_{L}$ and $E_{L}$ displayed the deteriorate of the relative $Nu_{x} Re_{x}^{ - 1/2}$.

## Final results and future guidance

The main purpose of this research is to discuss the behaviours of controlling parameters consisting of Prandtl–Eyring, permeable media, size of nano molecule particles, velocity slip, thermal radiative variable, Biot number, Eckert number, Reynolds number, and Brinkman number on the efficiency of the PTSC water pump MoS_2_–Cu/EO and Cu–EO hybrid/nanofluid. The efficiency of SWP is discussed, by making a decline in the flow of hybrid nanofluid MoS_2_–Cu/EO and single nanofluid Cu–EO, along with the description of heat transfer. The mathematical formulation is converted into ordinary differential equations by applying an appropriate similarity transformation. Then the system of converted ordinary differential equations is solved using Keller Box Scheme, and the extracted results can be described as follows:

- Only augmented the fluid velocity, whereas parameters reduce the fluid acceleration.
- The parameters increase the temperature of hybrid nanofluid MoS_2_–Cu/EO and single nanofluid Cu–EO.
- The entropy production increases when the effect of parameters is acting on the system. Besides, the effect of parameters reduces the rate of entropy generation.
- By uplifting the parameters of the skin friction coefficient was increased. However, parameters reduce the rate of skin friction coefficient.
- Increase in the parameters resulted in an increment of heat transfer rate, whereas the same variation was reduced due to the effect of Prandtl–Eyring and velocity slippery parameters.
- The thermal efficiency range of MoS_2_–Cu/EO over Cu–EO is from the minimal rate of and maximized up to.

For further investigation, additional factors such as the superior chemical and thermal criticality must be included in the flow behavior at large values of the thermal conductivity parameter. The determination of these two additional factors must be archived, in this further investigation.
